# NETQUANT: Automated Quantification of Neutrophil Extracellular Traps

**DOI:** 10.3389/fimmu.2017.01999

**Published:** 2018-01-15

**Authors:** Tirthankar Mohanty, Ole E. Sørensen, Pontus Nordenfelt

**Affiliations:** ^1^Division of Infection Medicine, Department of Clinical Sciences, Faculty of Medicine, Lund University, Lund, Sweden; ^2^LEO Pharma A/S, Ballerup, Denmark

**Keywords:** immunofluorescence, segmentation, quantification, single-cell analysis, NETosis

## Abstract

Neutrophil extracellular traps (NETs) that are extensive webs of DNA covered with antimicrobial proteins into the extracellular environment during infection or inflammation as a part of their defense arsenal. Image acquisition of fluorescently labeled NETs and subsequent image-based quantification is frequently used to analyze NET formation (NETosis) in response to various stimuli. However, there are important limitations in the present methods for quantification. Manual methods tend to be error-prone, tedious, and often quite subjective, whereas the software-rooted options are either semi-automatic or difficult to operate. Here, we present an automated and uncomplicated approach for quantifying NETs from fluorescence images, built as a freely available app for MATLAB^®^. It is based on detection of a set of clearly defined parameters, all related to the biological manifestation of NETs and allowing for single-cell resolution quantification and analysis.

## Introduction

Neutrophils play an important role in the host defense response against pathogens owing to their antimicrobial protein-rich granules (1), reactive oxygen species-production (2), and their ability to engulf pathogens through phagocytosis (3). More recently, neutrophil extracellular traps (NETs) have been identified as a novel defense mechanism in the neutrophil arsenal (4). NET formation (NETosis) is an active process and has been characterized as a novel mode of cell death (5). During NETosis, neutrophil DNA decondenses and is subsequently extruded into the extracellular environment with bound neutrophil granule proteins. Accordingly, decondensed DNA/chromatin, increased DNA staining area, and DNA with bound granule proteins are hallmarks of the NETosis process. Intact NETs, consisting of a DNA backbone embedded with neutrophilic proteins are essential for exerting biological effects during infection and inflammation. The DNA backbone has been demonstrated to be composed of either nuclear DNA or mitochondrial DNA (6). NETs are released in response to a variety of infectious agents, sterile mediators of inflammation, and endogenous host molecules (4). Although the end result of NETosis is the extrusion of DNA fibers coated with antimicrobial proteins, the overall phenotype and intracellular cell signaling pathways governing the process may differ and are dependent on the type of stimuli triggering NET induction (7). There has been a massive surge of interest in the field as several recent reports have indicated a link between NETs and a wide variety of morbidities, including autoimmune disease and thrombosis (8). Therefore, degrading or inducing NETs may represent novel treatment options in the future.

Detection of NETs using immunofluorescence in a sample typically involves two fluorescent channels, one channel depicting staining for the decondensed DNA and the other a NET-bound protein such as histone complexed with DNA or neutrophil elastase. The overlapping areas of staining observed after merging DNA and protein channels yields the total area under NETs. Hence, the number of NET-forming cells can be estimated by quantifying the combined overlapping areas of staining for DNA and NET-bound protein in an image (9). Manual methods are used frequently for quantification but have limitations in being error-prone, tedious, and subjective. Also, parameters that define a NET-forming event often cannot be uniformly applied across a large batch of images using manual methods. Automated image quantification, therefore, offers significant advantages, as many images can be processed quickly using stringent NET-defining parameters. Although methods have been published on microscopy-based automated NET quantification (9–11), the methods are either not fully automatic or are not easily operable without reasonable prior knowledge (10) and typically rely on an increase in the staining area as the sole criterion to define the extent of NET formation without information at the single-cell level. Therefore, an automated, approachable software based on single-cell analysis would greatly benefit researchers working with NETs.

Here, we present NETQUANT, an app for MATLAB that performs immunofluorescence image-based NET quantification and describe its implementation with the aim of delivering a user-friendly freely available tool. It is based on single-cell analysis and along with an increase in surface area, NETQUANT also considers deformation of the nucleus and an increase in DNA:NET-bound protein ratio as inclusion criteria to quantify cells undergoing NETosis or NETs that have already been extruded. Thus, NETQUANT may allow for unbiased NET quantification, with more stringent, biologically relevant, NET definition criteria that can be applied across different data sets rapidly and conveniently.

## Results

### Workflow for NETQUANT-Based Identification and Quantification of NETs

**Figure 1:**
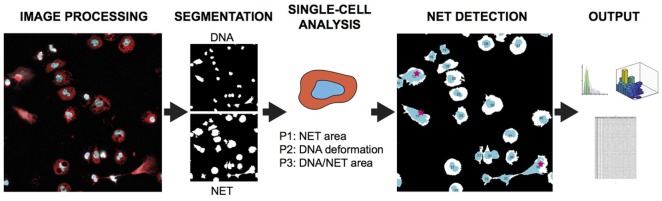
NETQUANT overview and testing. Basic outline of NET quantification process starting with two-channel image processing, followed by segmentation of cells, analysis of cell properties, detection of cells undergoing NETosis (red stars), and output of data.

The analysis principle is outlined in Figure 1 and can be fully automated with batch-processing after initial setup. The Bio-Formats framework (12) is implemented to allow users to read most microscope images and to standardize metadata presentation. The parameters adapt to different magnifications and resolutions through the registered pixel size in the image metadata. The software is based on the following general steps: (A) Preparation of data by reading and converting images to suitable format. (B) Segmentation of cells in both channels. (C) Analysis of identifiable cell properties. (D) Comparison of cell properties from the two channels for identifying NET-forming cells. (E) Output of results.

### Software Overview

**Figure 2:**
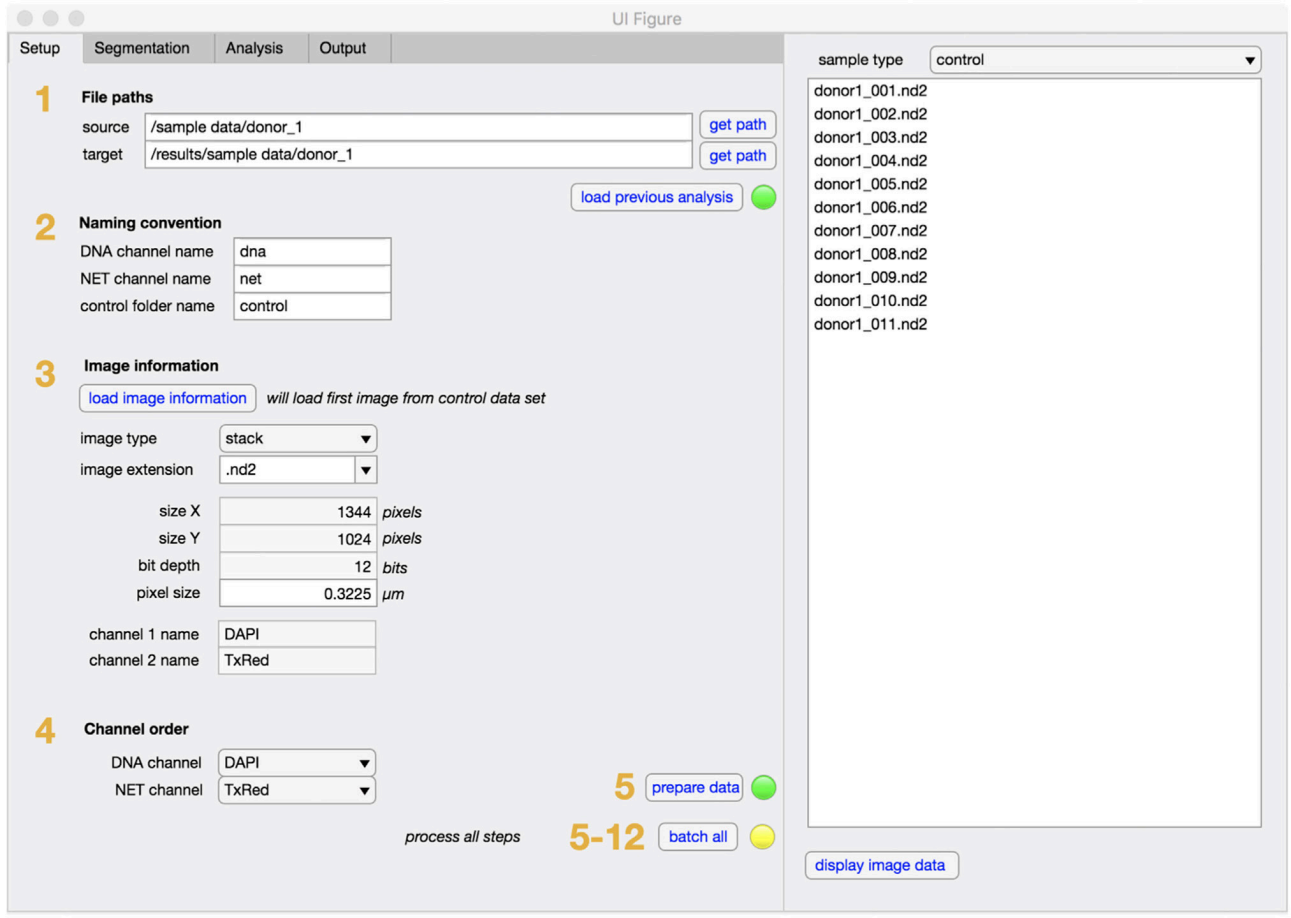
Setup tab for NETQUANT. File path of dataset(s) to be analyzed are entered into set up tab of the software and the file path for the data output is assigned (step 1). The naming conventions defining the channels (step 2), image information (step 3), and channel order (step 4) necessary for the analysis are to be provided by the user. Setting up all fields prior processing the datasets (step 5) is recommended to ensure image conversion in a standardized fashion.

NETQUANT is written as a standalone app in MATLAB and can easily be installed and used without prior knowledge of MATLAB. The graphical user interface (GUI) is divided into two main sections, one tab-based for setup, segmentation, analysis, and output of data, and one list-based to show and access the sample images (Figure 2). Each step of the NET-analysis process is clearly indicated with numbers so users can easily follow. There are batch options indicated for processing of multiple images (default) or for batching entire data sets (recommended after initial setup). In the setup tab, file paths (step 1), user naming conventions (step 2), image information (step 3), and channel order (step 4) are defined, before initial loading and processing of images is started (step 5). This ensures that the image data are converted and organized in a standardized way. The raw images are always left unaltered, and new copies are stored in the target folder along with processed images and results for easy access.

**Figure 3:**
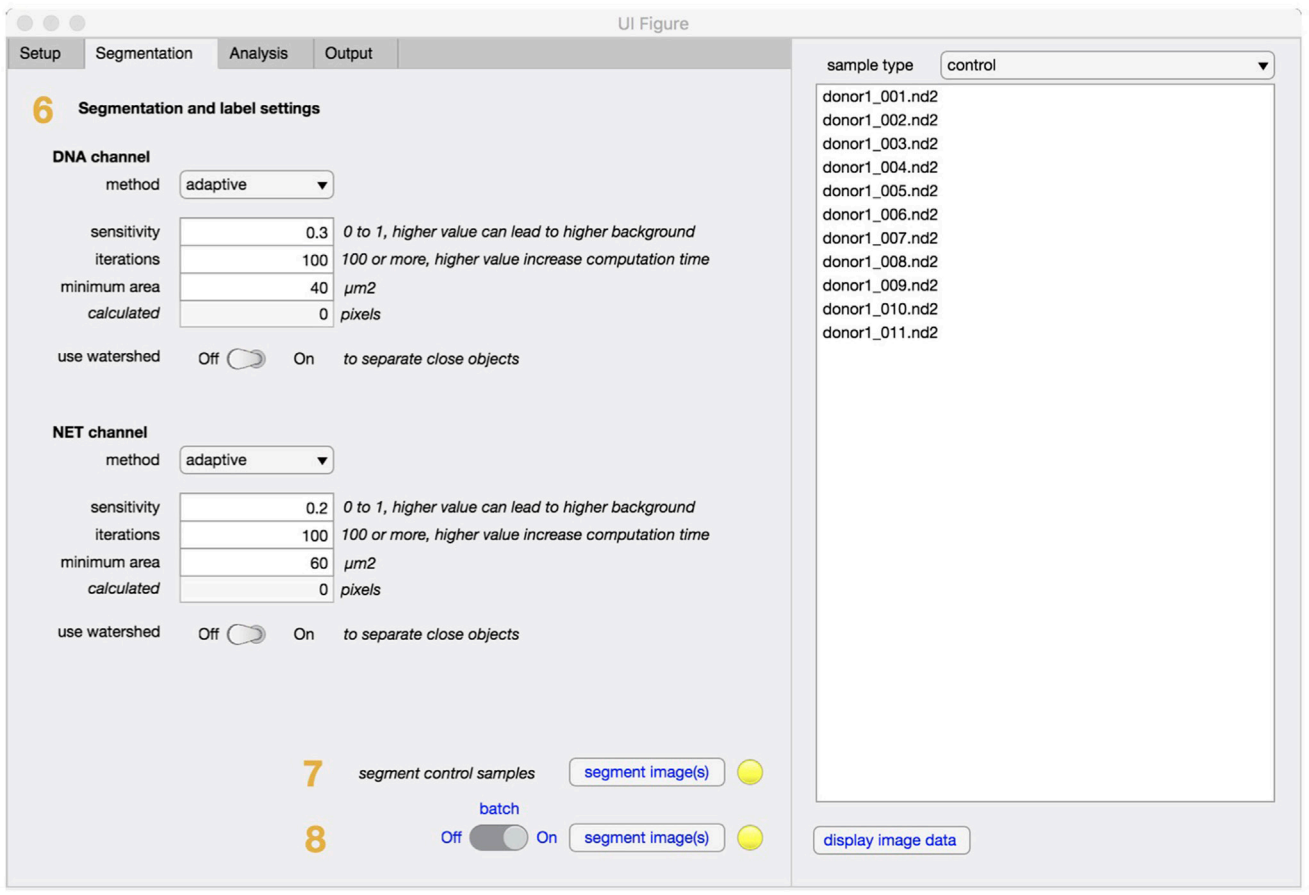
Segmentation tab for NETQUANT. The segmentation parameters used during analysis include method, sensitivity, iterations, minimum area of an unstimulated neutrophil (step 6). The use of adaptive segmentation is recommended and the settings provided can be left unchanged for most purposes. Control samples are first segmented (step 7), prior to stimulated samples (step 8).

In the segmentation tab (Figure 3), method and settings for identifying individual cells are defined (step 6). In most cases, these settings can be left unchanged. If the software is applied to other cell types the minimum cell area might need to be adjusted to filter out unwanted regions. The segmentation will fully adapt to objective and camera, based on the information retrieved in the setup step and an equivalent number of pixels to minimum cell area will be automatically calculated and displayed. Control samples with non-stimulated cells are always required and will be segmented (step 7) before any stimulated samples are processed (step 8).

**Figure 4:**
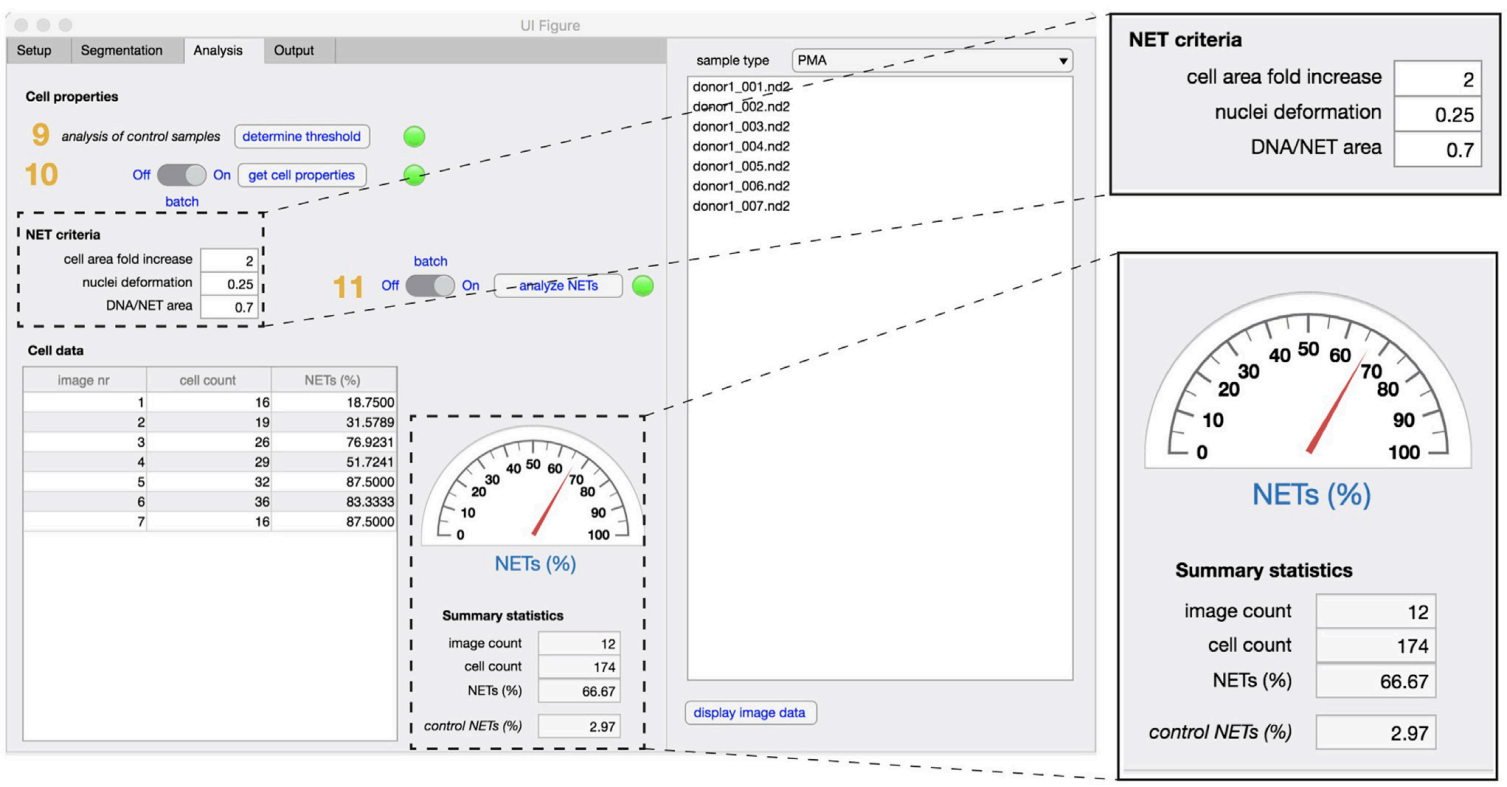
Analysis tab. The properties that define unstimulated cells are analyzed first in non-stimulated control samples (step 9), followed by analysis of cell properties in experimental samples (step 10). To identify NETs, the three criteria are defined [fold increase in the cellular area, extent of nuclear deformation (from 0 to 1) and DNA/NET area] and applied in both control and stimulated samples (step 11). Enumeration of NET formation in both samples are displayed in the summary statistics section. The total number of images used and the total cell count in the datasets is also displayed.

In the analysis tab (Figure 4), the properties of identified cells are first analyzed and compared to non-stimulated control samples (steps 9,10). Based on the defined NET criteria (see below), the number of NET-positive cells is determined (step 11) and displayed along with image and cell counts and corresponding control values.

**Figure 5:**
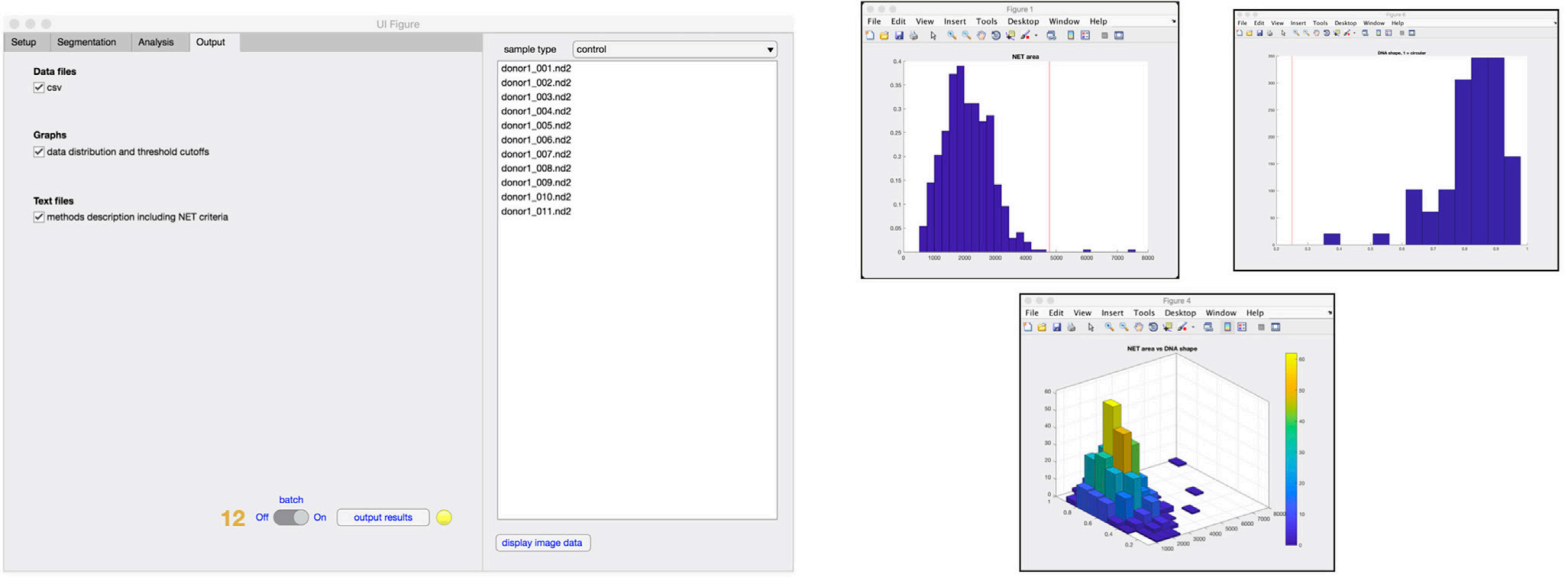
Outputs from analysis. Results from the analysis can be generated in the form of .csv file containing data from individual cells and histograms representing the distribution of cells undergoing increase in the area, nuclear deformation and DNA/NET ratio (step 12). A 3D graph of NET area versus nuclear deformation is also available.

Finally, in the output tab (Figure 5), the user selects what type of data that will be stored in the results folder, including comma-separated value (.csv) files, data distribution graphs (Figure 5), and method description text file with corresponding NET criteria values (step 12). At any time of the process, an image can be selected from the sample list and the image displayed along with the analysis that has so far been undertaken.

### Characterization of NETs Using Three Biologically Relevant Criteria

NETs were defined based on three biological parameters, NET area, DNA deformation, and DNA/NET area (P1–P3, Figure 1) by using information from both DNA and NET channels. The algorithm requires a control data set of non-stimulated neutrophils from the same experiment for calibrating the size and shape as captured by the microscope. This makes the software more robust to differences in optical properties of the microscope and the way images are acquired. Based on known properties of NETs (13), a NET-positive cell is defined as either covering a larger area (P1), having decondensed DNA (P2, measured as deformation of DNA circularity), or having an increased DNA/marker protein co-localization (P3, measured as DNA/NET marker area ratio), as compared to the control data set. The P2 parameter is based on measuring the circularity of cell DNA staining, *C*_DNA_, defined as following,

(1) $$C_{\text{DNA}}=\frac{4\cdot \pi \cdot A_{\text{DNA}}}{P_{\text{DNA}}^{2}},$$

where *A*_DNA_ is the area of DNA staining and *P*_DNA_ is the perimeter of the cell DNA staining. The parameter thresholds are set by the operator, and recommended ranges are indicated in the GUI of the software. It is possible for the operator to set parameter thresholds that may inflate the numbers of identified NETs, but since the results are always presented in the context of the control cells, it would also inflate the proportion of control cells positive for NETs, thus reducing the risk for user-induced artifacts. We recommend that the percentage of positive control cells is always reported along with experimental results.

### Software Evaluation

To evaluate the efficacy and accuracy of NETQUANT, three separate sets of fluorescent images (*N* = 2,244 cells) of control versus PMA-stimulated cells were analyzed either using NETQUANT or analyzed manually by an experienced operator. The images were acquired under normal conditions without any bias, to test the software performance by a previously inexperienced user. This also meant the presence of poor quality images and subsequent handling of these by the software were also evaluated.

**Figure 6:**
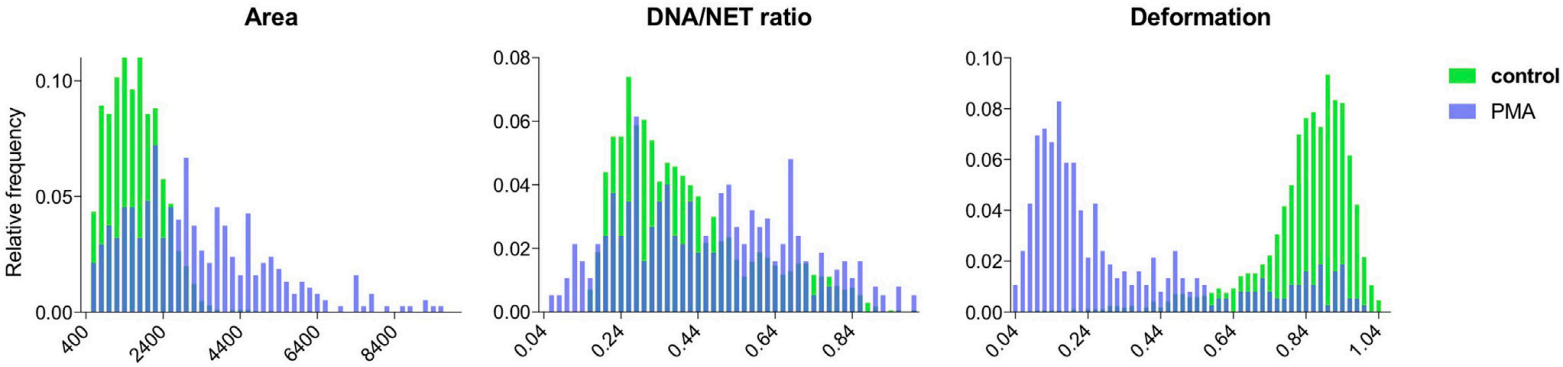
Comparison of NET formation in control and PMA-stimulated neutrophils using NETQUANT. Image datasets from non-stimulated or PMA-stimulated neutrophils were analyzed using NETQUANT. The outputs generated from NETQUANT analysis describing changes in the cell area, nuclear deformation and DNA/NET ratio from control (green) and PMA-stimulated neutrophils (blue) were compared. This type of data are easily available within the app for any analyzed sample. Data are from three independent experiments.

Both the control data and the NET-induced samples were successfully segmented and the properties of the segmented cells were analyzed. For the control sets, cells had normal distributions of area size, DNA deformation, and DNA/NET marker ratio (Figure 6). The NET-induced samples were clearly different with shifted distributions of the NET parameters (Figure 6). All of these cell properties are available as output for individual cells and might be used to provide more detailed discrimination of cells undergoing NET formation, such as separating cells with decondensed nuclei with those that have undergone full NETosis.

**Figure 7:**
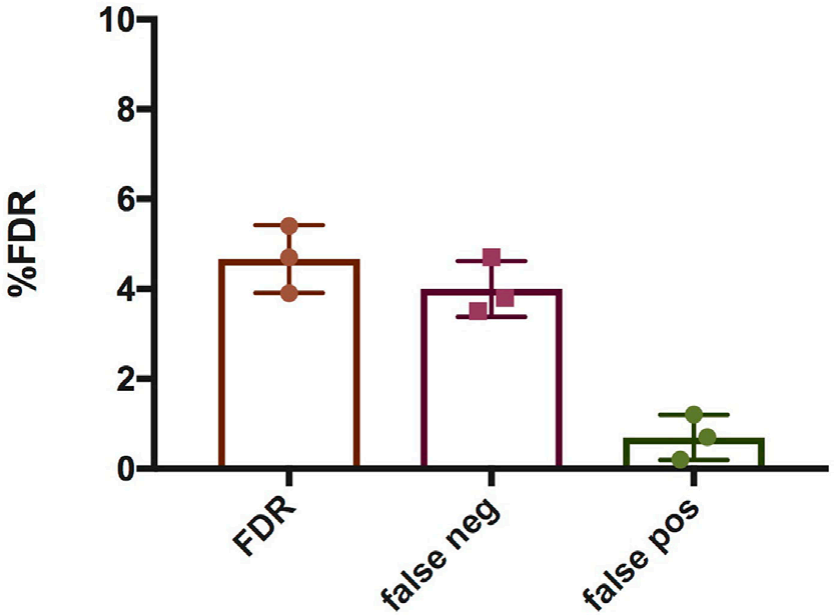
False discovery rate (FDR) associated with NETQUANT. Bar graphs indicating FDR associated with NETQUANT. NETs were quantified by NETQUANT or manually by an experienced user and were compared to assess FDR. The total FDR was found to be 4.7 ± 0.75% (mean ± SD), with false negatives 4 ± 0.63% and false positives 0.7 ± 0.5%. Data are from three independent experiments.

**Figure 8:**
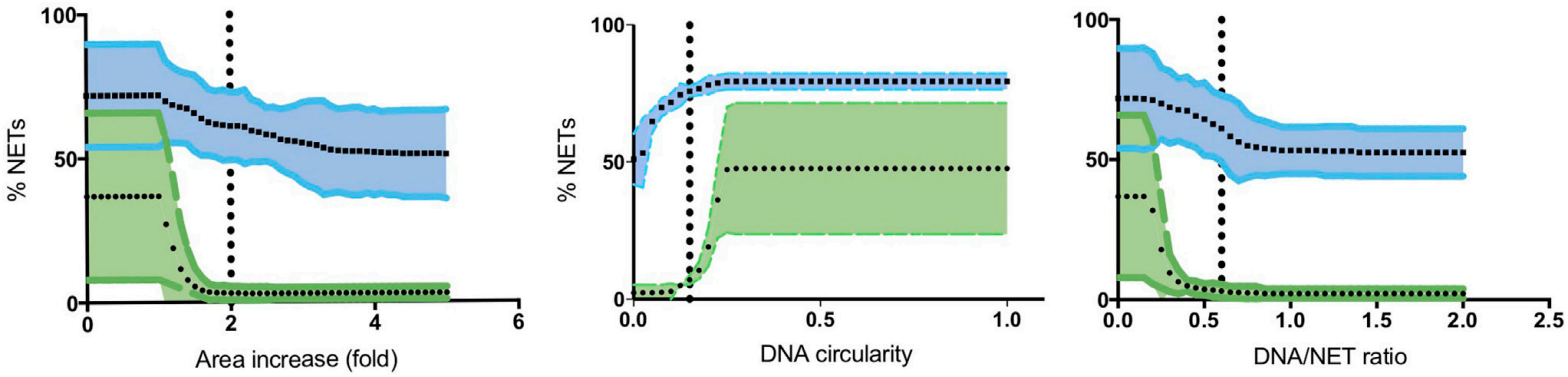
Optimization of NET criteria and reduction of false discovery rates (FDR). FDR could be reduced by modifying the NET criteria. Simulations of varying each parameter was performed 50 times, while keeping the other two at a constant value (indicated with vertical lines). Mean values with 95% confidence value in shaded area are shown for both stimulated (blue) and non-stimulated controls (green). The criterion with the highest resolving power was the DNA/NET area parameter.

**Figure 9:**
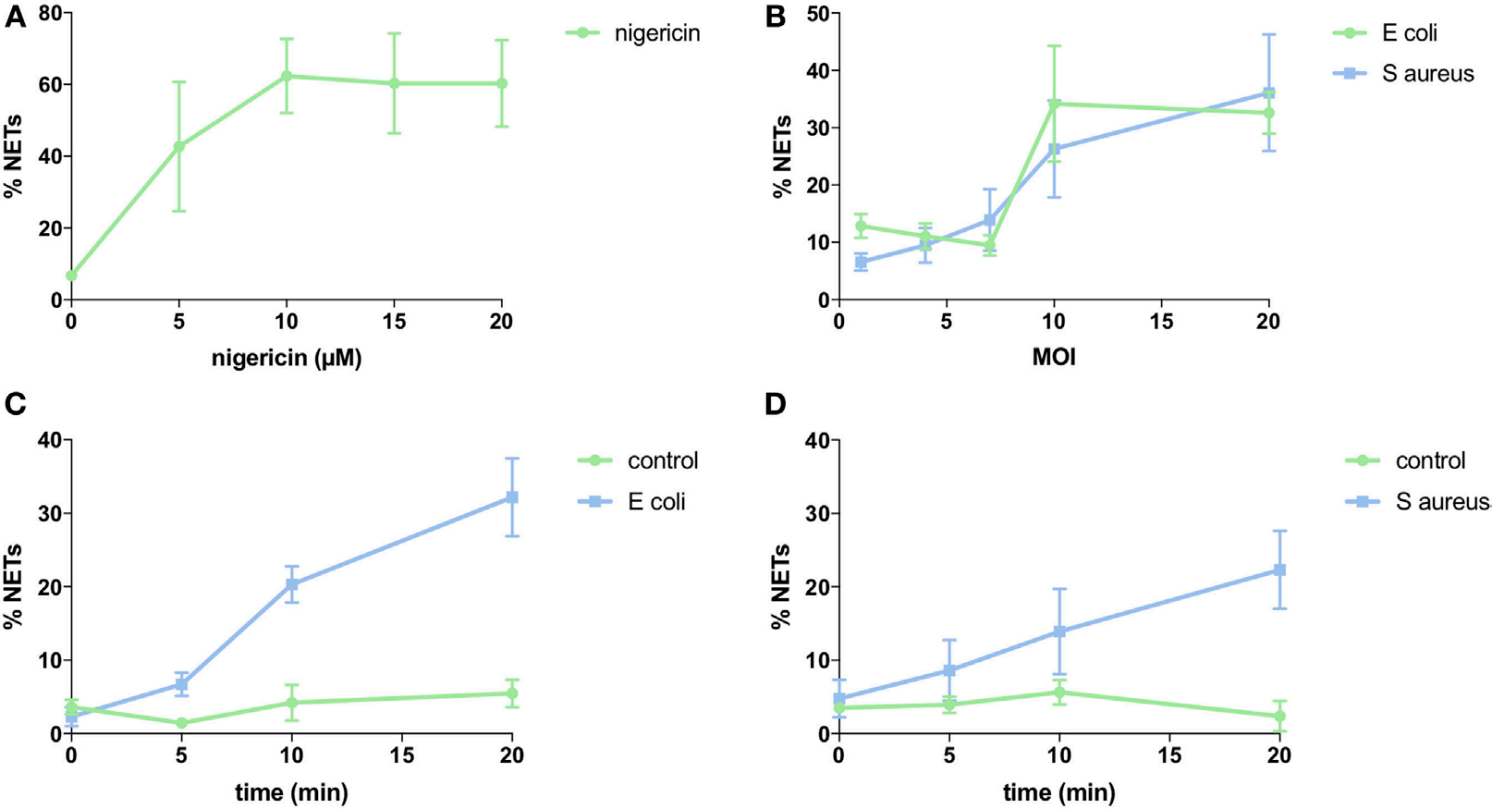
NET induction with nigericin and bacteria stimulation. Analysis of NET formation with various stimuli and using neutrophils from three different donors (SEM is shown). **(A)** Dose-dependency of nigericin stimulation. Three separate experiments with 2,893 cells analyzed. **(B)** Dose-dependency of bacterial stimulation. Three separate experiments with 3,177 cells analyzed for *Escherichia coli* and 3,636 cells analyzed for *Staphylococcus aureus*. **(C)** Time-dependency of bacterial stimulation at MOI 10. Three separate experiments with 3,573 cells analyzed. **(D)** Time-dependency of bacterial stimulation at MOI 10. Three separate experiments with 3,751 cells analyzed.

To quantify NETs, the three NET parameters were set empirically within the recommend range as it would be by the user upon initialization. Once set, all analysis was performed in an automated manner, and for similar samples (same imaging and preparation method), the parameters can typically be left unchanged. This resulted in accurate detection of most NETs (Figure 7) with an average false discovery rate (FDR) of 4.7%, with a false positive rate of 0.7%. Of note, when using the three factors independently, many NETs are typically missed, showing the strength of multiple criteria for single-cell analysis. Simulations show that it is possible to further reduce the FDR by optimizing the parameters (Figure 8), and this will depend on usage scenarios. When comparing data sets taken at 20× (0.75 numerical aperture) versus 40× (0.95 numerical aperture) magnification, the results were comparable without any changes in the settings, indicating that the software can adapt successfully. The result outputs are as csv-files with single-cell data and graphs of data distributions. To simplify method reporting, an option to generate a methods description text file is also incorporated. In order to verify that the software could quantify NETs in a broad range of scenarios, various known NET-inducing stimuli where tested in a dose- or time-dependent manner (Figures 9A–D). The stimuli were the ionophore nigericin, the Gram-positive bacterium *Staphylococcus aureus*, and the Gram-negative bacterium *Escherichia coli*. The bacteria were tested both with dose (multiplicity of infection, MOI) and time (at MOI 10). In total, over 17,000 cells were analyzed across experiments with three separate donors. Figures S1–S3 in Supplementary Material show representative images from the analysis. Taken together, NETQUANT appears to automatically handle large datasets and accurately identify NETs in a broad range of scenarios.

## Discussion

In comparison with other forms of NET measurements, image-based analysis of NETs has the theoretical advantage that it can directly detect NET-forming cells, and that it can be done on a single-cell level. This can help in eliminating background signal from events not related to NETs. However, a drawback of image-based analysis is that is time-consuming and can be subjective, and can be difficult to automate for single-cell analysis. Here, we examined NET formation in stimulated neutrophils on glass cover slips using an automatic approach for rapid and reliable quantification of NET formation on a large batch of cells using NETQUANT. Currently, fully automated image quantification software dedicated for quantifying NETs as presented in the manuscript are unavailable to researchers.

The machine-learning approach to identify segmented regions as NETs presented by Coelho et al. (10), offers some advantages over previous methods, but has limitations in the accessibility of the software to non-technical users, and in the effort needed for training the software for new conditions. Additionally, although this approach appears to adequately assess the overall NET formation in a sample, it does not provide any information about the activation status of single cells or nucleus deformation brought about by chromatin decondensation.

The approach used by NETQUANT has similarities to a previous published semi-quantitative method of NET quantification based on ImageJ functions (9). The method described by Brinkmann et al. is reliable but due to its semi-automatic nature requires several manual steps prior to analysis, including manual threshold determination (with risk of user bias), and also does not provide single-cell information. Another recent approach uses semi-automated acquisition of confocal 3D volumes, coupled with ratiometric whole image analysis of extracellular DNA and neutrophil markers (11). They show that by capturing the whole signal from the three-dimensional NET structure, they achieve markedly increased sensitivity and could identify small increases in NET induction. Although very simple, and thus likely robust, this approach does not provide single-cell information—which is the highest sensitivity possible.

NETQUANT is advantageous as it has been developed specifically for quantifying NETs with high stringency, has integrated all algorithms into a simple app, automatically adapts to differing image and sample conditions, and outputs detailed single-cell data, allowing for more advanced post-analysis of NET formation. Because the software is automatic, multiple images can be processed rapidly and may also be used for high-throughput analysis.

Previous quantification approaches utilized an increase in cellular surface area as the sole criterion for NET formation. NETQUANT is able to detect NET formation based on increased surface area, but also considers nuclear deformation associated with NETosis and the shift in the DNA/NET-staining ratio, increasing the resolving power of the analysis. Thus, cells that have underwent NET formation can be distinguished from cells that have underwent only nuclear decondensation due to a lower DNA:NET ratio. The NET definition criteria included are user-friendly and can be easily modified to compensate for variation in cell characteristics. The variation in the NETs detected is directly linked to the internal control samples and this allows for comparable image analysis from data generated by multiple users and conditions. There exist different pathways for NET formation (14), particularly those induced by PMA and bacteria or ionophores, respectively (15). If there is a big different in morphology, such as lytic and non-lytic NET formation, this could be a potential limitation for image-based quantification. However, our results show that NETQUANT can successfully identify NET formation from both main categories of NET-inducing stimuli, most probably since NETQUANT rely on multiple parameters for NET identification.

Our analyses reveal that the software identifies most cells undergoing NETosis, and most importantly has a very low false positive rate. Simulations show that the FDR can be further reduced by the user by adjusting the NET definition criteria provided in the software. Future versions of the software could include automatic optimization of NET parameters and other improvements that might be suggested by users.

NETQUANT is an easy-to-use tool that can quickly and automatically accurately detect cells that are positive for NETs. We believe this will become a useful tool for the entire NET community, as well as for diagnostic purposes in clinical settings.

## Methods

### Neutrophil Isolation

One volume of 2% Dextran in 0.9% NaCl was mixed with one volume of blood collected in heparin tubes and the sample was allowed to sediment at room temperature. The samples were centrifuged, resuspended 0.9% NaCl, which was layered on top of Lymphoprep (Axis-Shield) as described previously. Erythrocytes were lysed by resuspension in sterile water and washed in 0.9% NaCl. The cells were counted and resuspended in RPMI-1640 medium with 2 mg/ml HSA.

### NET Induction on Coverslips

Coverslips were washed once with PBS, followed by incubation in 12-well plates with 0.01% poly-l-lysine. Coverslips were washed once in PBS neutrophils were added to each well. The cells were allowed to adhere at room temperature, followed by incubation at 37°C in 5% CO_2_. Neutrophils in RPMI with 2 mg/ml HSA were used as non-stimulated controls. NETs were induced by the addition of 20 nM PMA (Phorbol 12-myristate 13-acetate, Sigma) for 150 min or nigericin at a concentration of 5, 10, 15, and 20 µM for 3 h in RPMI with 2 mg/ml HSA.

### Bacterial Culture and NET Induction

*Staphylococcus aureus* (clinical strain 050701) and *Escherichia coli* (clinical strain) were plated overnight on Todd Hewitt (TH) agar plates for 6–8 h at 37°C in presence of 5% CO_2_. Bacteria were inoculated in 10 ml TH broth medium and cultured overnight at 37°C with 5% CO_2_. 0.5 ml of the overnight culture was transferred to 10 ml of TH broth at 37°C with 5% CO_2_ and cultured for 3 h. The bacteria were washed thrice in PBS. For NET induction the bacteria were added to neutrophils in RPMI with 2 mg/ml HSA adhered to poly-l-lysine coated coverslips as described above. Neutrophils were exposed to bacterial MOIs of 1, 4, 7, 10, and 20 for 1 h to assess NETosis. A MOI of 10 was used to plot the time course for NETosis at 0′, 5′, 10′ and 20′. Samples were processed for immunofluorescence as described below. Between 1 and 8% NET-positive cells were seen in the control samples.

### Immunofluorescence Microscopy and Image Acquisition

The images were prepared using standard NET protocols (16), with two-channel staining of DNA and NET-associated proteins, such as elastase and myeloperoxidase (17, 18). After stimulation of neutrophils, the medium was removed and the coverslips were washed once in PBS. Cells were fixed with 4% paraformaldehyde, followed by washing with PBS. Cells were permeabilized by addition of 0.5% Triton-X-100. Cells were washed PBS, followed by incubation in blocking buffer (5% goat serum in PBST). Cells were incubated in primary antibody against human neutrophil elastase for 1 h at 37°C and washed with PBS. Secondary Fab fragment labeled with Alexa594-labeled secondary antibodies (Molecular Probes) raised in goat against rabbit diluted to 1:1,000 in blocking buffer was added for 1 h at 37°C, followed by washing in PBS.

Coverslips were mounted using PROLONG Gold anti-fade reagent with DAPI (Life technologies). Slides were dried in the dark at room temperature overnight before examination of PMNs and NETs using fluorescence microscopy. Images were acquired using a Nikon Ti-E equipped with a Andor Zyla 4.2 CCD camera or a Hamamatsu Orca CCD camera, using Plan Apochromat 20× and 40× objectives. NIS-elements 5.1 (Nikon) software was used for image acquisition and processing.

### Image Segmentation and Cell Identification

**Figure 10:**
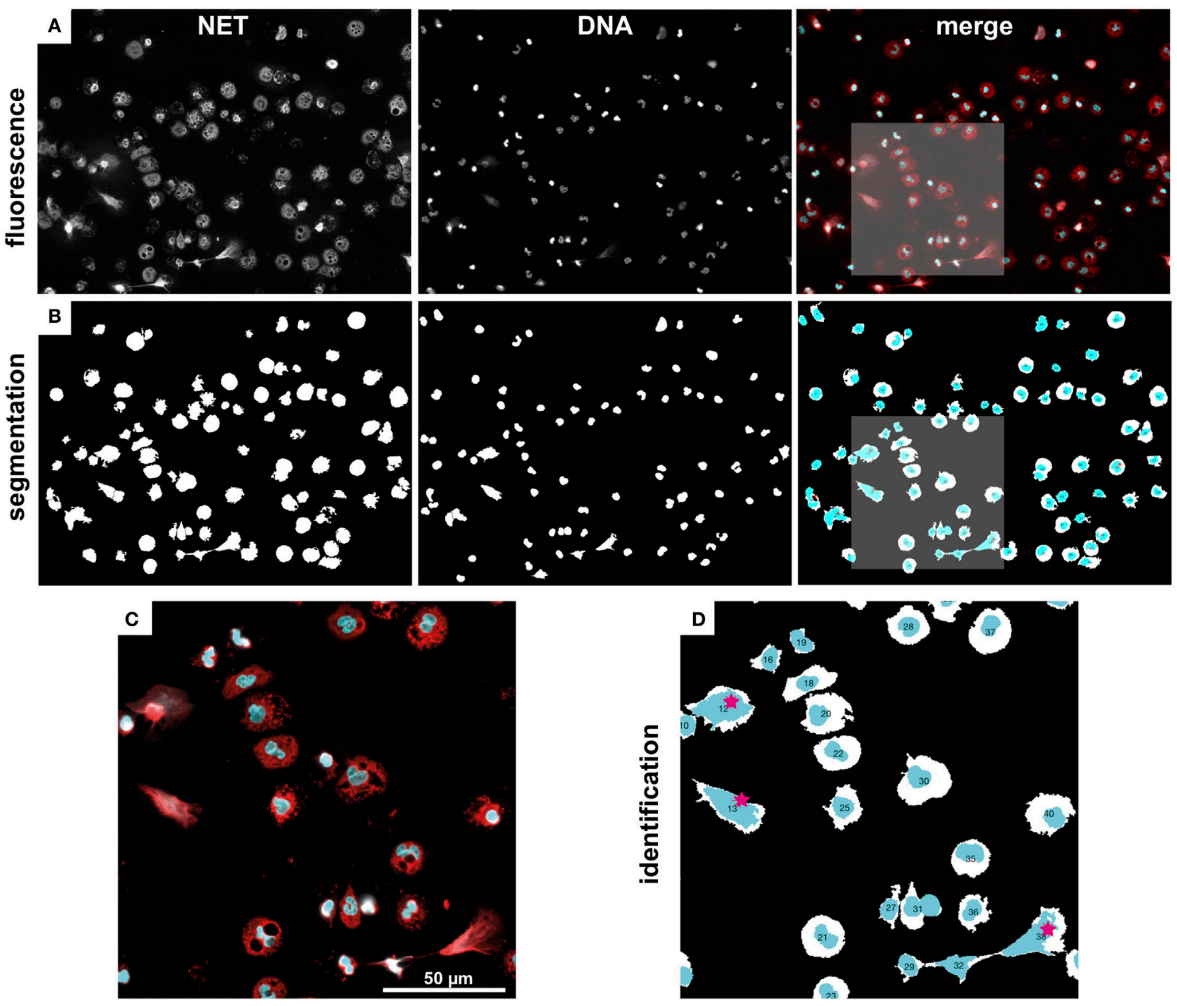
Image segmentation and NET labeling. Representative example of immunofluorescence images, segmentation, and NET identification. **(A)** Fluorescence images of NET and DNA marker, as well as a merged color image (NET, red, DNA, cyan). **(B)** Segmented regions from fluorescence images in **(A)**. **(C)** Zoom-in from marked region in **(A)**. **(D)** Zoom-in from marked region in **(C)**. Automatically identified NETs are labeled with red stars. Note, the watershed separation of cells option was used in this example.

NETQUANT includes four options for segmentation of fluorescence images (Figure 10A) into distinct regions [Adaptive segmentation (19), Global segmentation (20), Active contour-based segmentation, either Edge (21) or Chan–Vese methods (22)]. The default algorithm, adaptive threshold-based segmentation, outperformed the others in all cases and was, therefore, used throughout all analyses. As compared to traditional threshold-based segmentation (Global option), which applies a single value to the whole image, adaptive segmentation computes a local threshold value for each pixel based on first-order statistics of the neighborhood. The ensuing matrix of local threshold values is then applied to the whole image, making it possible to adapt for uneven illumination or large differences in fluorescence intensity (Figure 10B). The other options are still included as their might be user scenarios where those approaches might be useful.

The segmentation algorithms will identify regions that contain cells, but can typically not differentiate between close or touching cell regions. NETQAUNT handles this through a watershed transform algorithm (23), which treats the image as a surface, with bright pixels representing elevation and dark pixels representing low ground. By then finding “basins” or “ridges” in the image, cells can be separated where they touch, based on their neighborhood connectivity in those pixels (see close cells in Figures 10C,D). As cells undergoing NETosis tends to cover large areas, the likelihood for them touching neighboring cells increases, and we, therefore, recommend using the watershed option activated in the software for very dense cell populations, but otherwise leave it off.

Once the image has been segmented into cell regions, each cell is color-coded and labeled with a number (Figure 10D) and saved as a new image. This allows for post-analysis of single cells, including data curation and sub-population analysis.

### Software Availability

The software is written as an app for MATLAB (MathWorks, Inc., USA). It is compatible with Windows, Macintosh and UNIX-based systems. A manual is available to guide users (see Supplemental Material). Installation file and sample data are available at nordlab.med.lu.se.

## Ethics Statement

Neutrophils were collected from healthy donors in accordance with the Declaration of Helsinki and approved by the ethics committee of Lund University (2013/728).

## Author Contributions

PN and OS initiated the study and algorithms were designed by TM and PN. Programming was done by PN and experiments by TM. Analysis was performed by TM. Manuscript was drafted by PN and TM with input from OS. All authors read and commented on the final manuscript.

## Conflict of Interest Statement

OS was employed by LEO Pharma A/S. All other authors declare no competing interests.
