# Supplementary figures and images for: NETQUANT: Automated Quantification of Neutrophil Extracellular Traps
Source: Front Immunol. 2018 Jan 15;8:1999. doi: 10.3389/fimmu.2017.01999 (PMC5775513; doi:10.3389/fimmu.2017.01999)

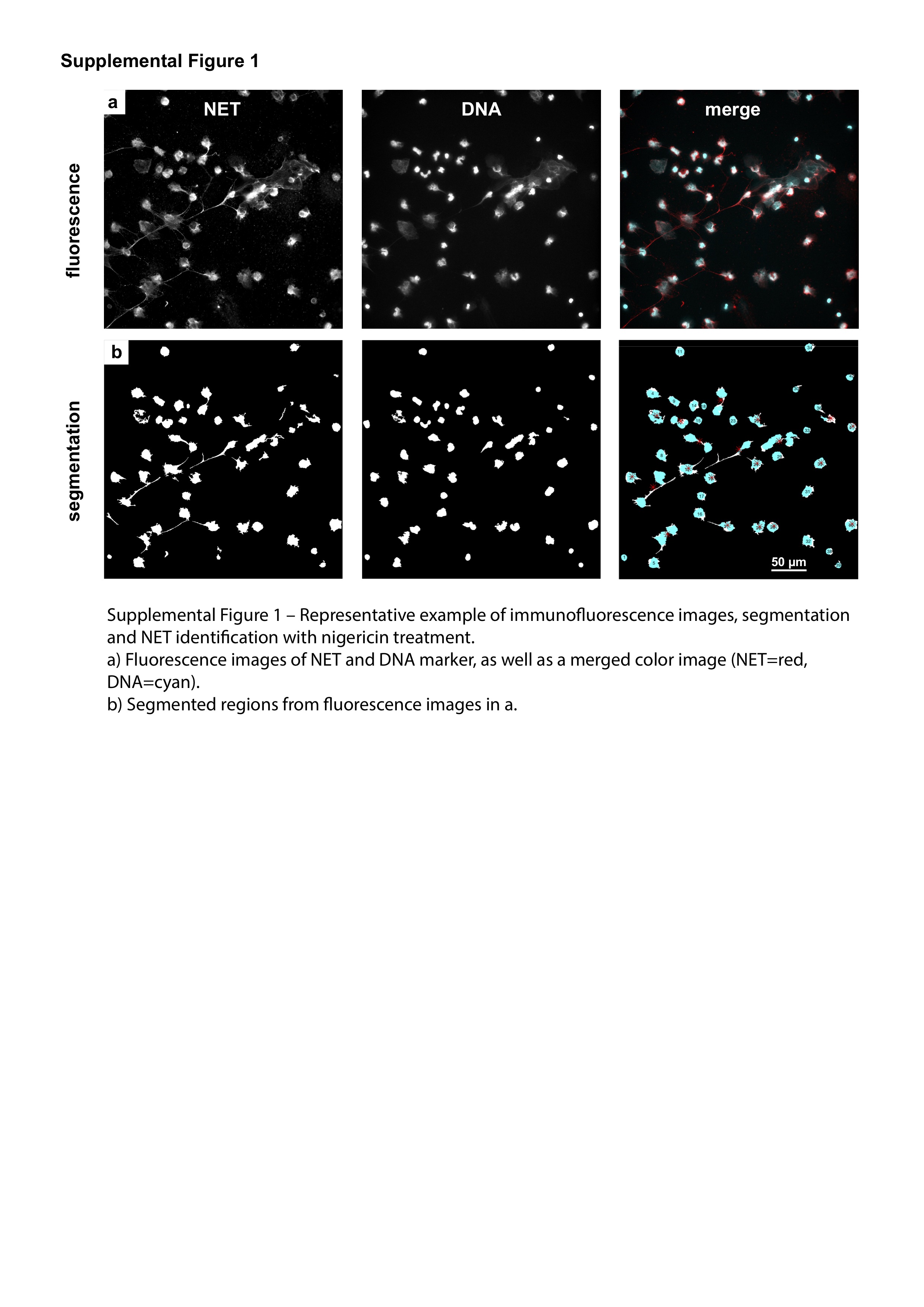

Supplement: Supplementary file 1 [file image_1.jpeg]

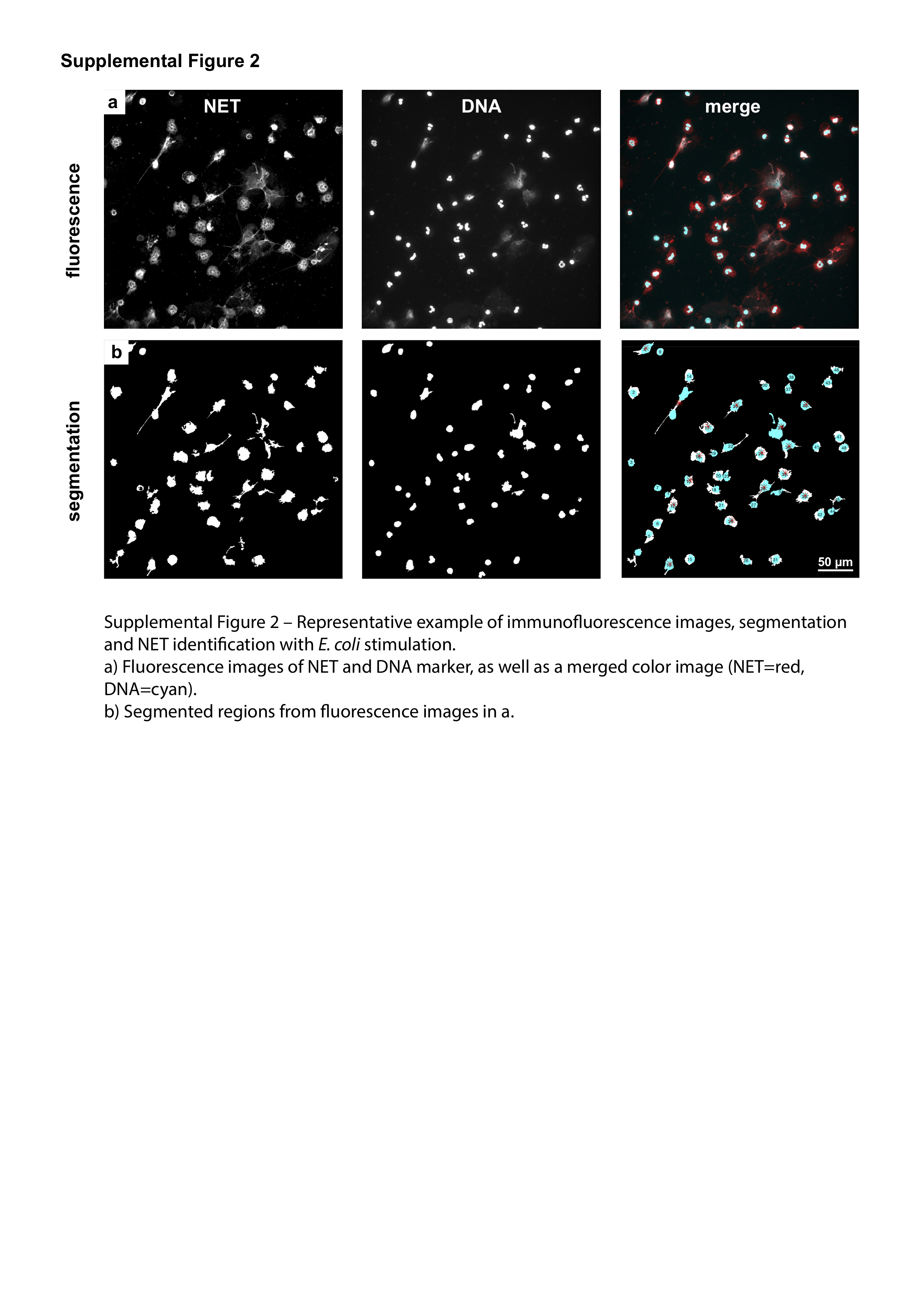

Supplement: Supplementary file 2 [file image_2.jpg]

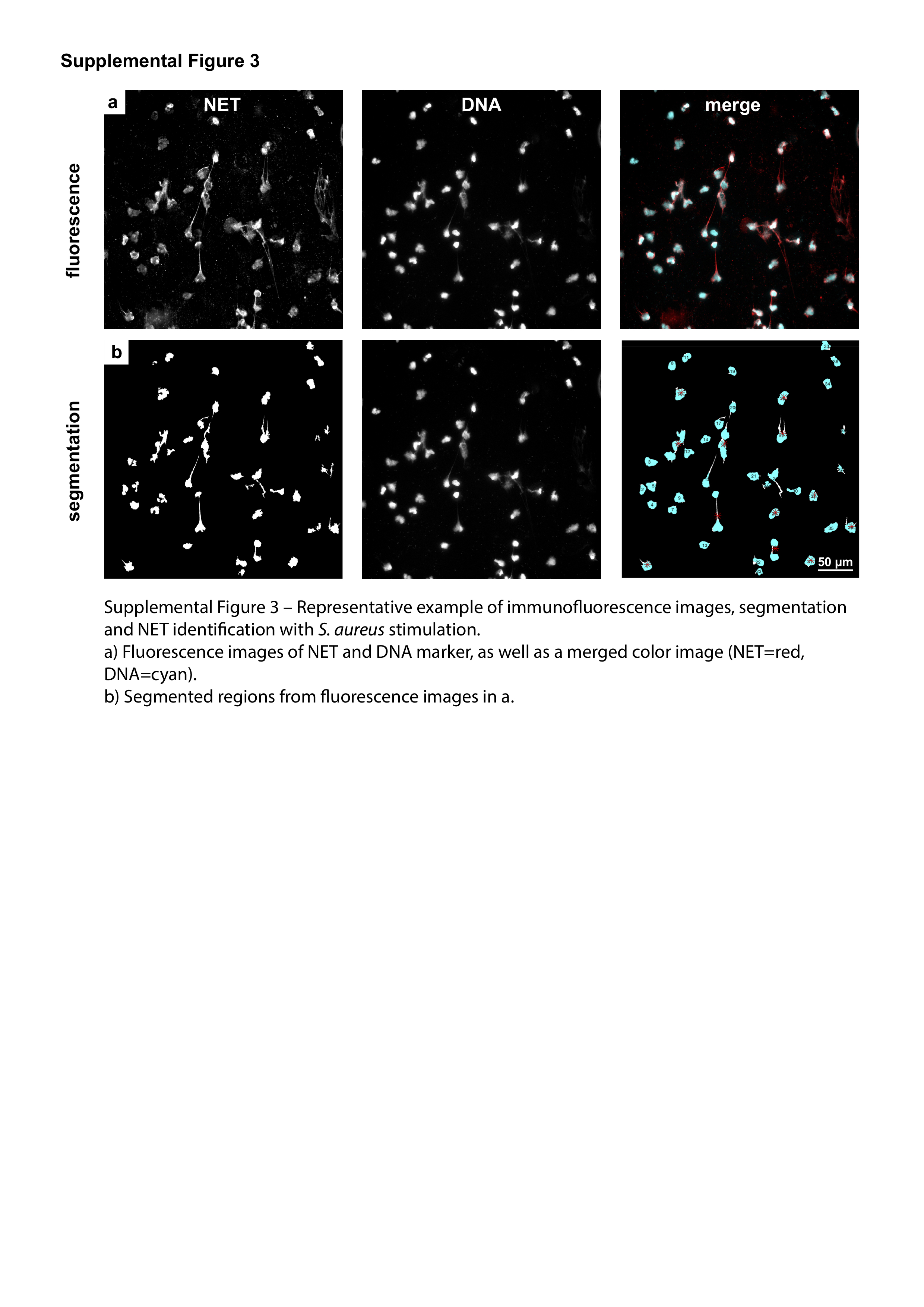

Supplement: Supplementary file 3 [file image_3.jpg]
