# Supplementary material for: NETQUANT: Automated Quantification of Neutrophil Extracellular Traps
Source: Front Immunol. 2018 Jan 15;8:1999. doi: 10.3389/fimmu.2017.01999 (PMC5775513; doi:10.3389/fimmu.2017.01999)
Supplement: Supplementary file 4 [file presentation_1.PDF]

NETQUANT software user manual version 1.1.

Version history:

Version 1.1. December 2017

- New features
  - Added option to set different segmentation settings for the two channels.
  - Changed the definition of P2 to relate to DNA staining circularity for improved NET detection.
- Bug fixes
  - Fixed problem with control folder being placed in the wrong order in some cases.
  - Removed threshold folder from sample list when loading previous analysis.
  - Fixed problem with batch processing when no cells where found in a sample. It is now being correctly handled as zero cells in the aggregate as well.

Version 1.0. September 2017 – Release candidate sent for review

1 Installation

1.1 Operating system and pre-requisite software

The software currently has been tested on Microsoft Windows 10 or macOS Sierra 10.12 and requires MATLAB 2016a (MathWorks).

1.2 Installation of software

To install the software, double click the download installer found on the website. Once installed, the NETQUANT appears as an icon in the application menu (app menu) of MATLAB and can be accessed easily by selecting it.

1.3 Optional download of sample data and demonstration video

Sample data used for test analysis can be downloaded from the software web site. Additionally, a demonstration video is available that will run the sample data step-by-step through the NET quantification procedure.

2 Use of software

2.1 Sequence import

To begin analysis of the raw data, file sequence/folder (**step 1**) to be analysed are imported into the *source* option. The processed data generated post-analysis is saved in a different folder in the *target* folder option. All Bio-Formats approved image formats (>140) are supported by NETQUANT.

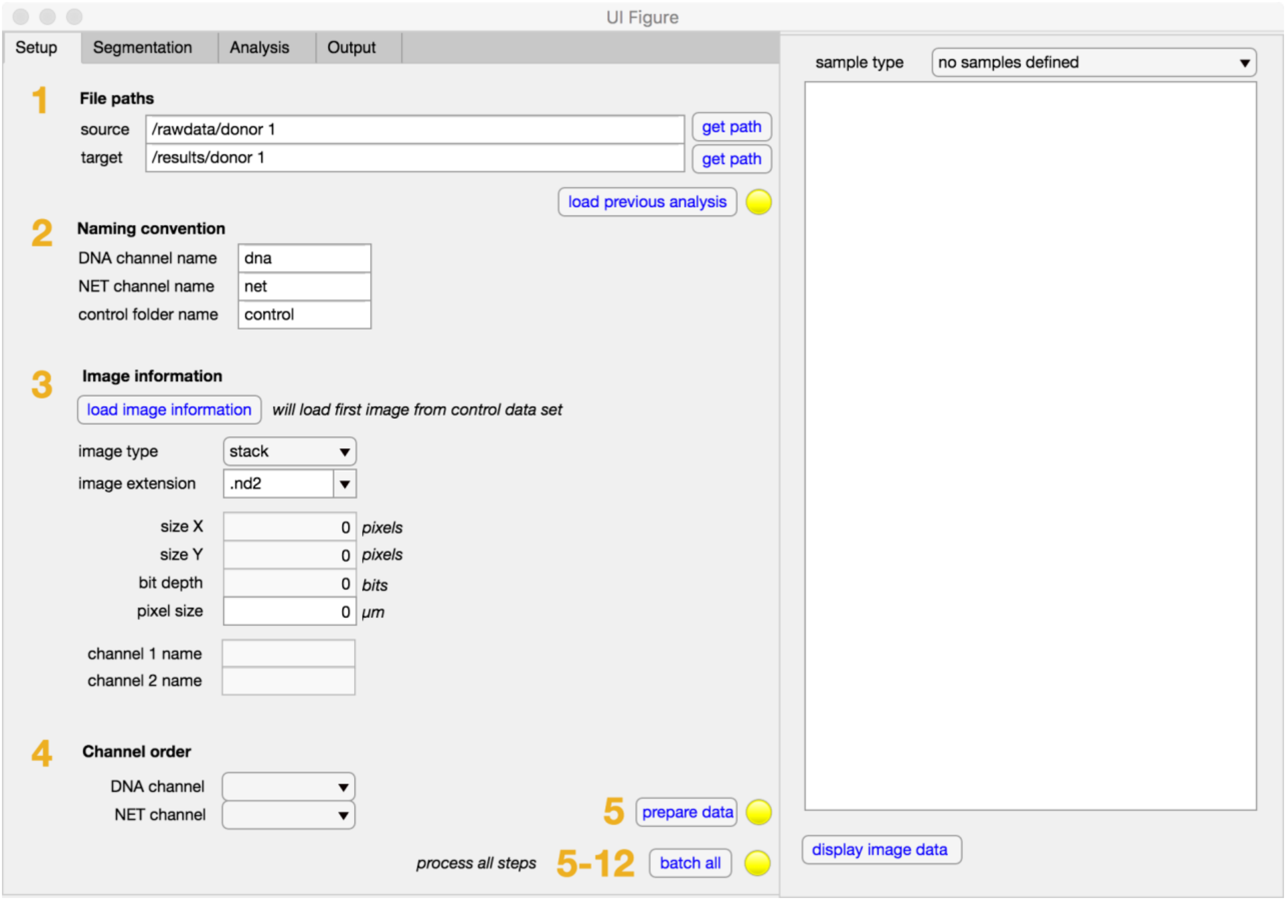

2.2 Naming of image channels to be analysed

The naming convention for the channels are to be decided by the user (**step 2**). NETQUANT performs NET analysis using 2 channels for DNA and NET-associated protein, namely -

DNA channel depicting staining for DNA.

NET channel depicting staining for NET-associated proteins such as neutrophil elastase, histones or myeloperoxidase.

Control folder depicts raw data from non-stimulated control neutrophils that are to be used as internal control for the experiment. The name can be changed by the user, but we recommend naming the control samples to be analysed as ‘control’ for ease of use. The control data is an essential pre-requisite for the analysis to function properly.

2.3 Setting image parameters/information for quantification

Image parameters can be fed to the software by clicking on the load image information button in the menu (**step 3**). NETQUANT extracts information such as image type, image extension, image properties and naming order of the channels from the first image in the control folder.

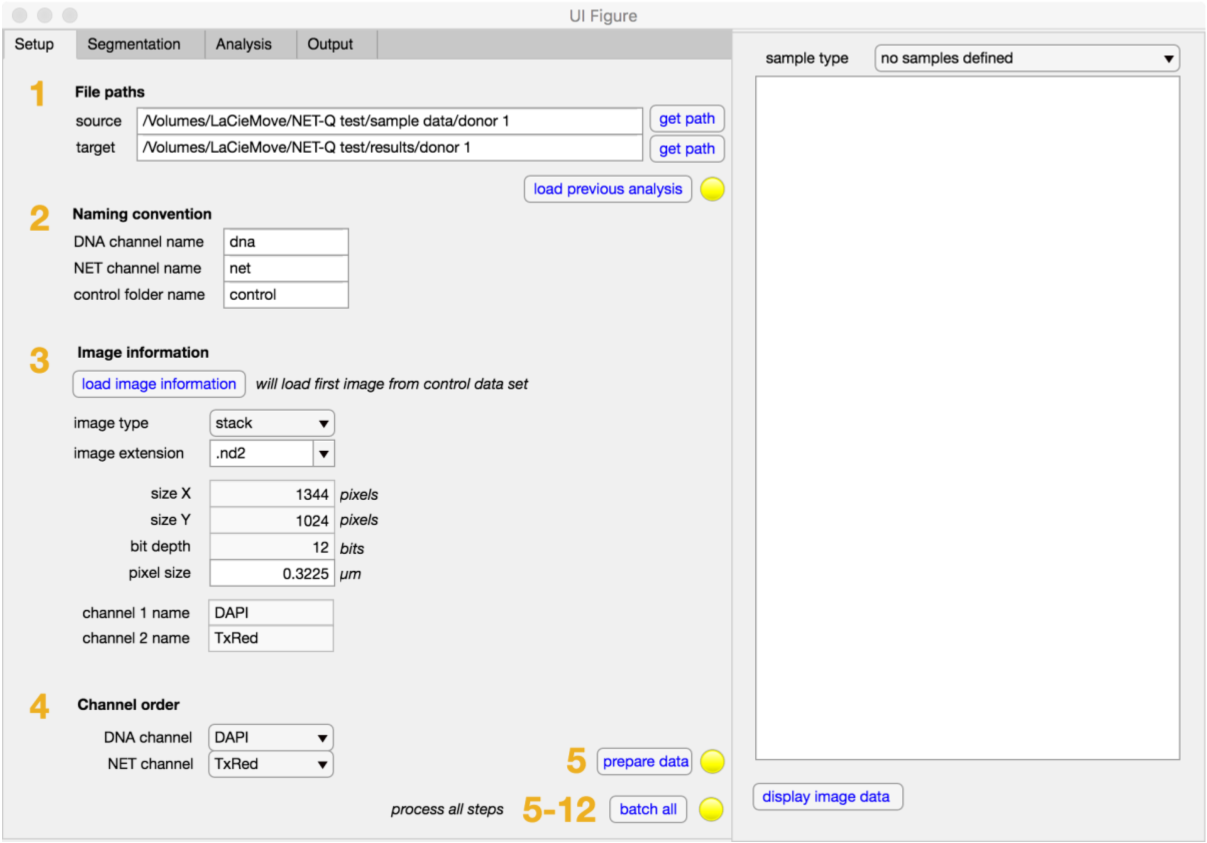

2.4 Selecting correct channel order

This option (**step 4**) is a ‘fail safe’ that has been included for the user in the software to prevent accidental mismatches in the naming of the image channels and staining for either the DNA or NET-associated protein.

2.5 Preparation of data for control and stimulated samples

The acquisition of primary image properties of the raw data is then acquired by clicking the *prepare data* button (**step 5**). The defined data sets appear as controls and the stimulated samples in the *sample type* menu. There is an option to automatically perform steps 5-12 (*batch all*).

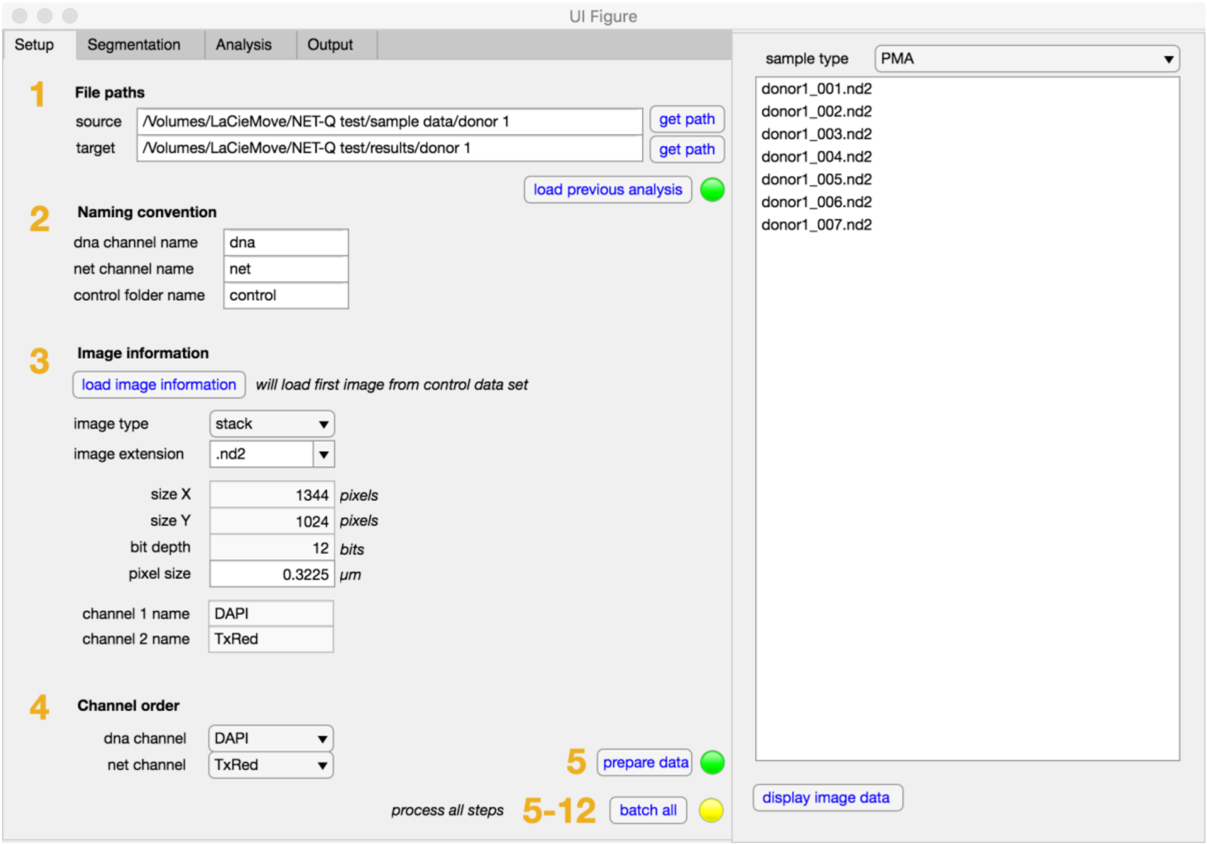

2.6 Setting parameters for image segmentation

Select data set to be segmented (**step 6**) in the *sample type* menu. The default pre-set on NETQUANT is to use adaptive segmentation and defines unstimulated neutrophils to have a minimum area of 50µm^2. The user can also manually adjust minimum area of an unstimulated cell, depending on the cell type.

We recommend using the watershed option for distinguishing closely placed cells to aid in image segmentation.

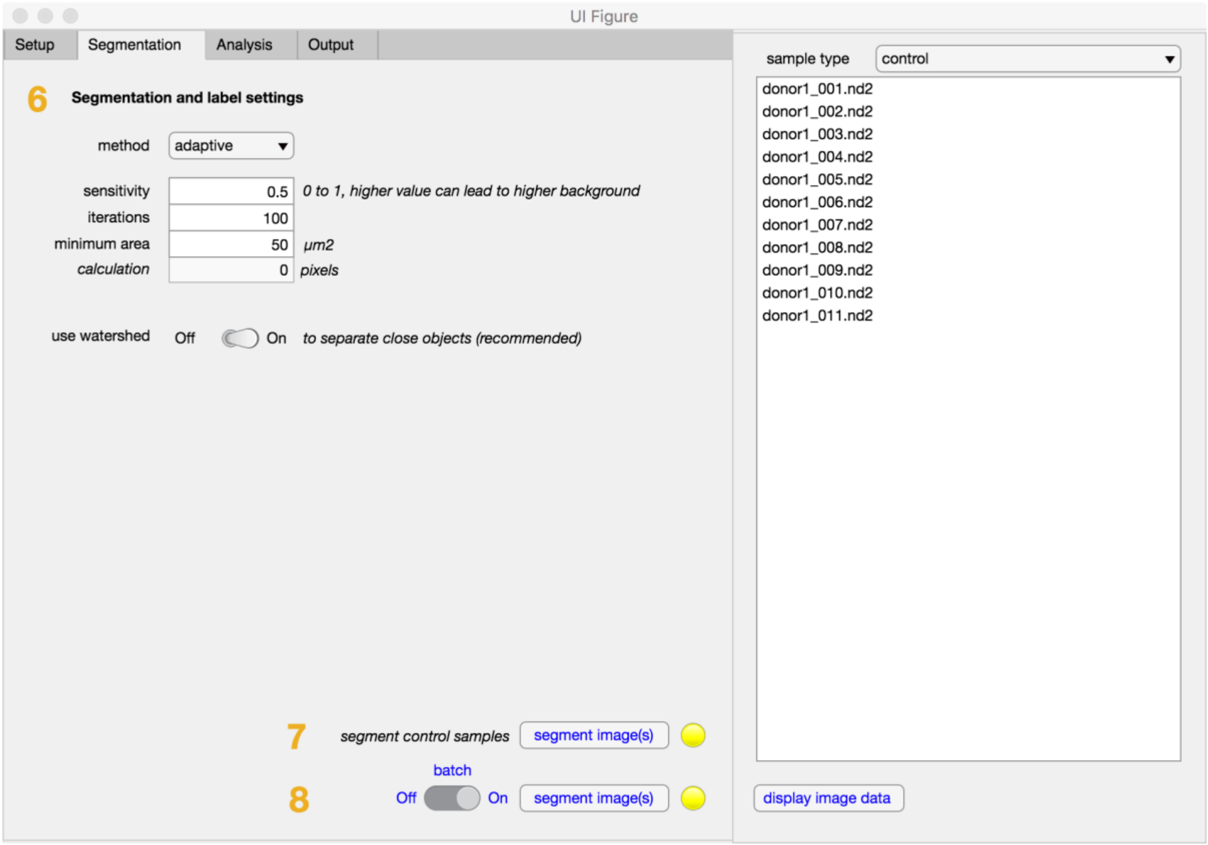

2.7 Segmentation of control images

The control images are to be defined first (**step 7**). The software automatically loads images from the *control* folder. Click on segment image(s) to initiate the process. Once, the process is complete, the user is also presented with the option to check if the segmentation is satisfactory and has resulted in the correct identification of cells by clicking the display image data.

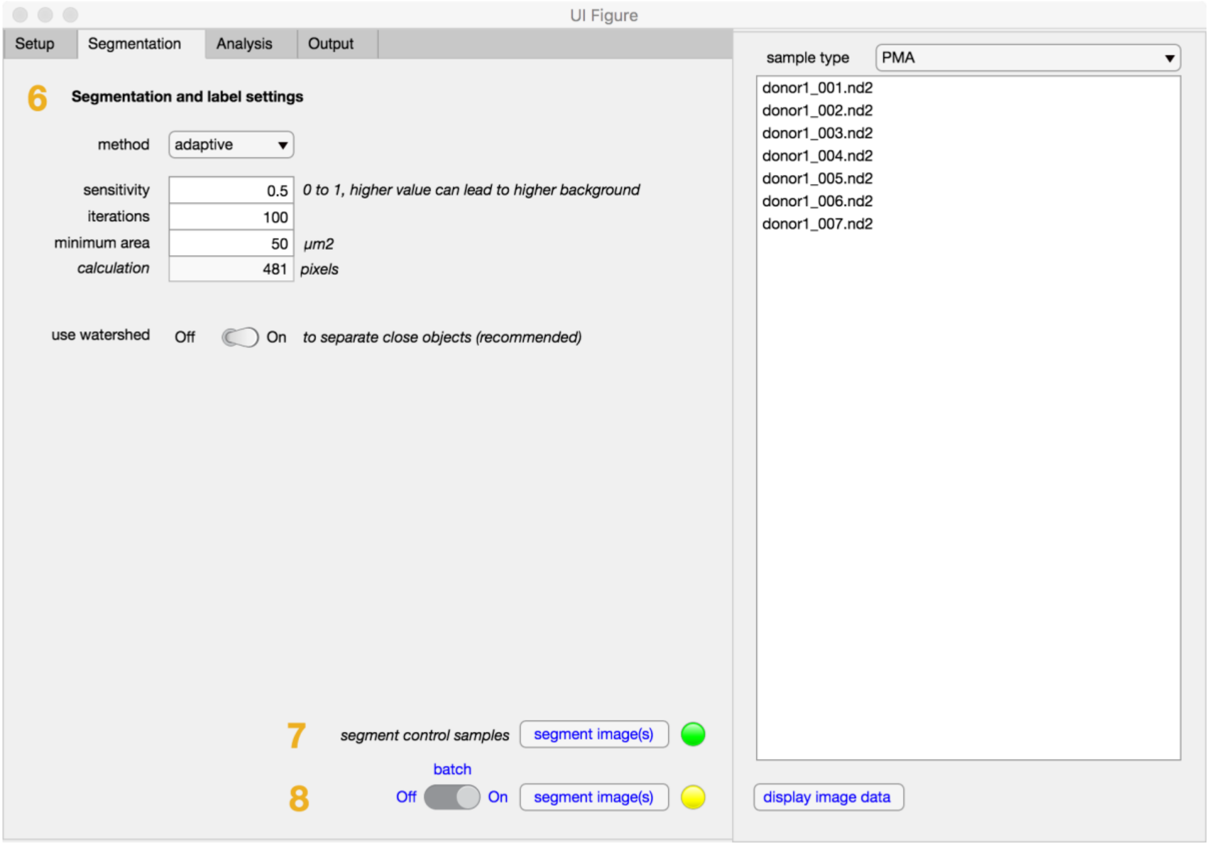

2.8 Segmentation of experiment images

The segmentation is then extended (**step 8**) to the experimental data sets (such as PMA) by selecting in the *sample type* menu. The *batch* option indicates whether to segment all images (recommended) or only the selected image.

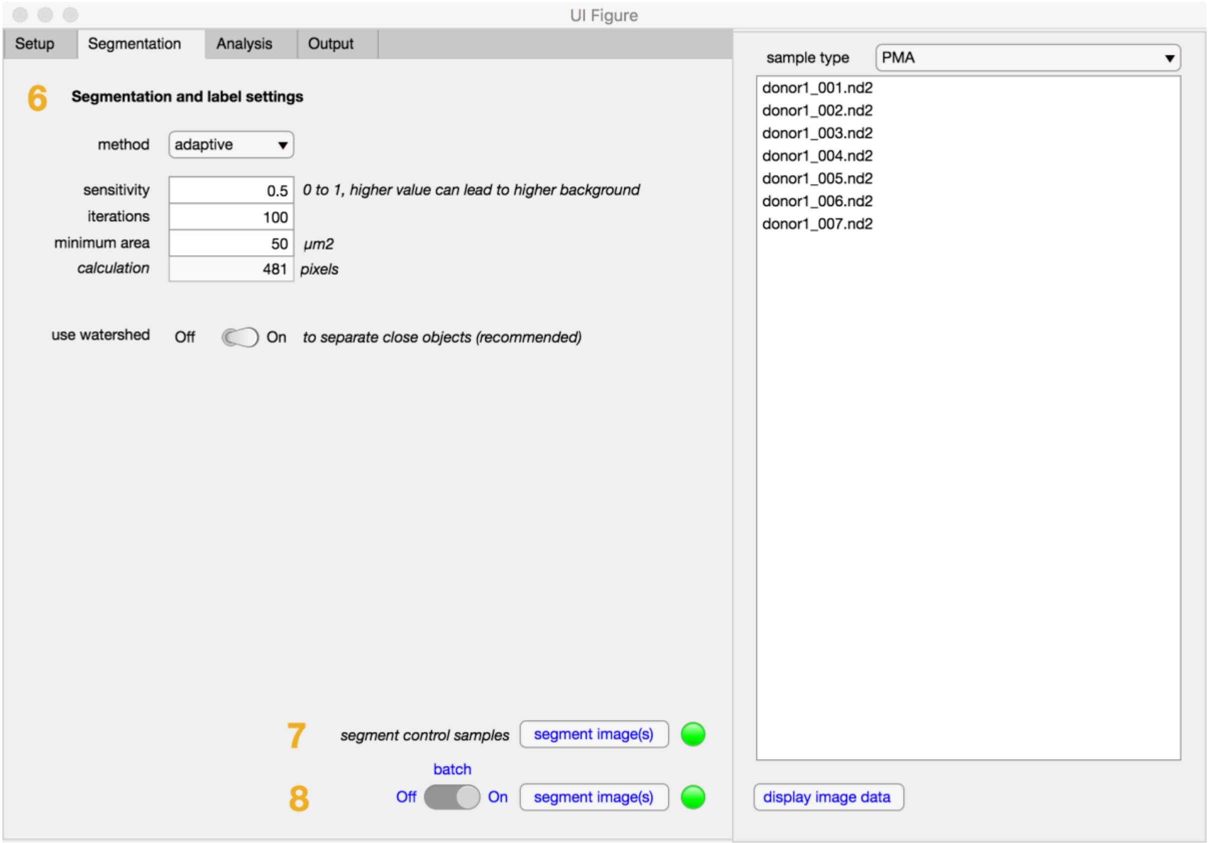

2.9 Determination of threshold in control samples

This step (**step 9**) gathers the individual cell properties from control data. The software automatically loads images from the *control* folder. Click on determine threshold to initiate the process.

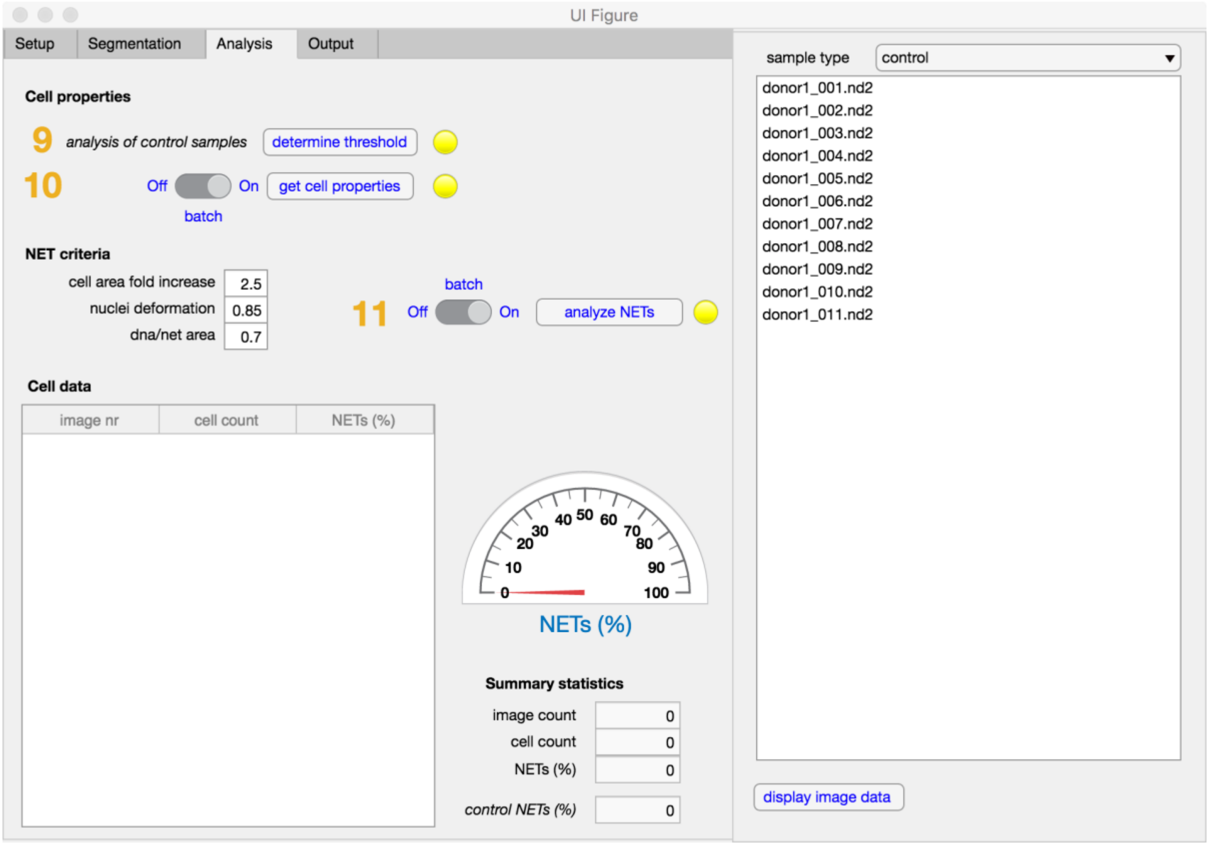

2.10 Determination of cell properties in experimental samples

This step (**step 10**) gathers the individual cell properties from experimental data that is selected in the *sample type* menu. The *batch* option indicates whether to analyze all images (recommended) or only the selected image.

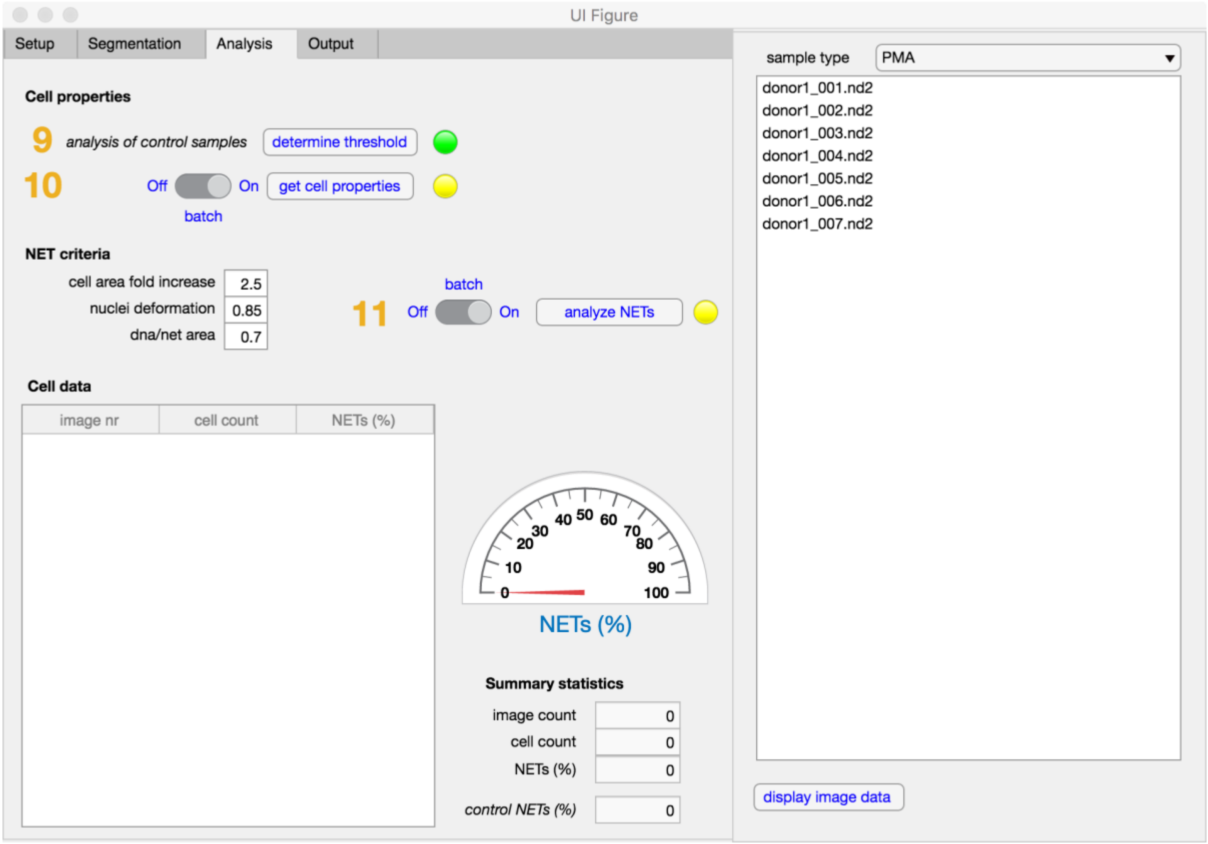

2.11 Defining NET criteria and analysis of NET formation

The pre-set criteria to define NETs in NETQUANT can be adjusted manually by the user to yield optimal results for a given sample. NETs are then analysed by clicking the *analyse NETs* button (**step 11**). The analysis can be performed on either the entire batch of images by selecting complete data sets (default) in the sample type menu or single images by clicking a single image within a data set, and deselecting batch processing.

The data are displayed as percentage NET formation and individual data points are presented in the cell data table. The *summary statistics* output below the gauge depicting NET-formation displays the total numbers of images and cells in the sample, along with the percentage of NET-formation in control samples.

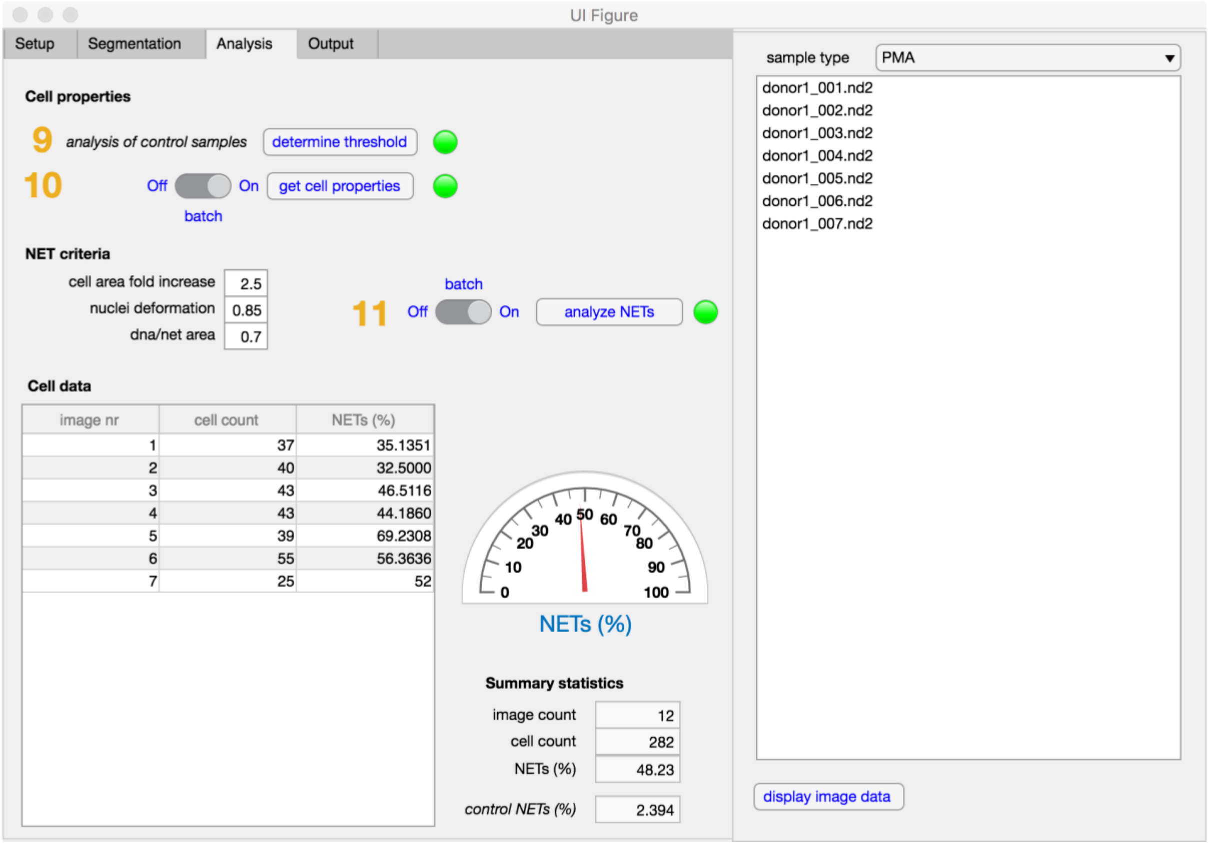

NET criteria can be adjusted by the user to optimize results. However, this has a direct effect on the number of NET-forming events detected during the analysis. Therefore, any changes in NET criteria are applied simultaneously to the controls and displayed in the summary statistics as illustrated in an example below.

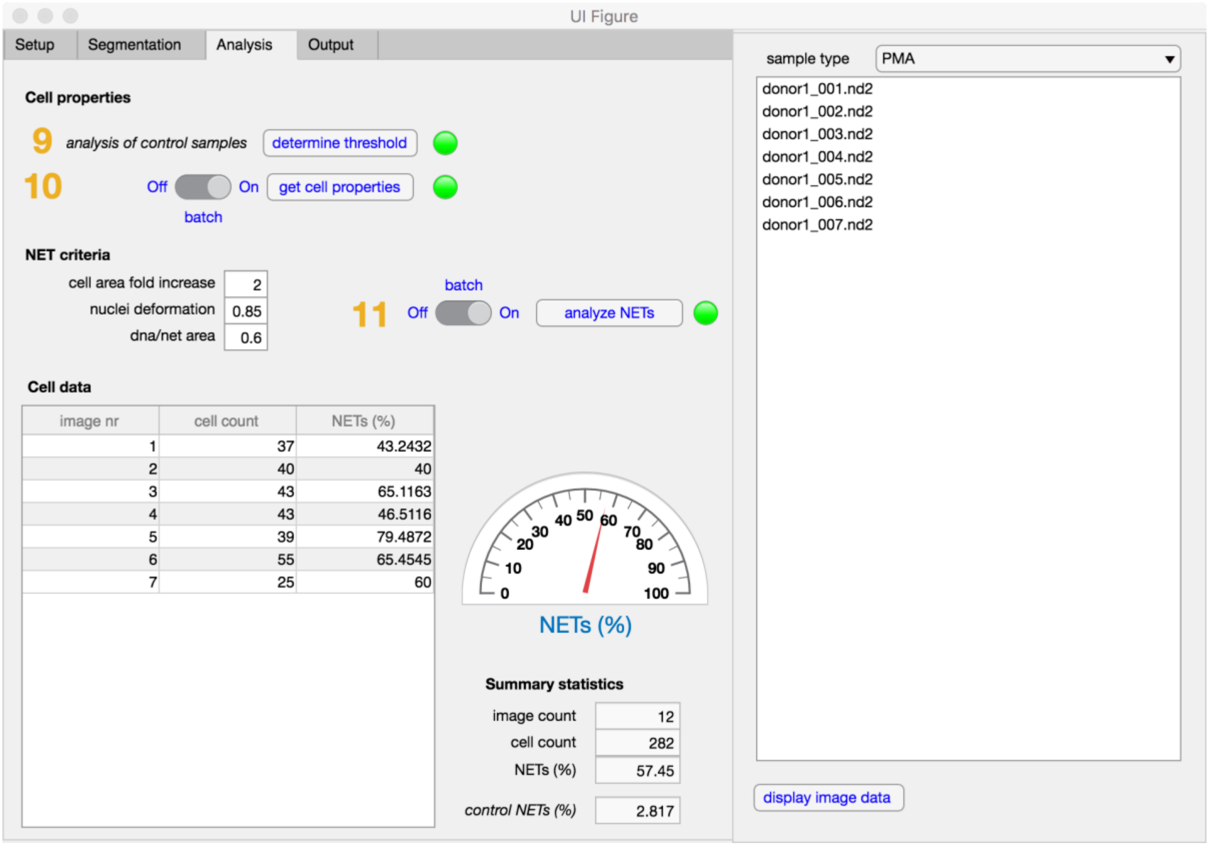

2.12 Data output files

NETQUANT has several options for displaying the image data generated from the analysis. The complete data set or an individual data point is selected from the *sample type* menu. The outputs can be obtained by simply ticking the dialogue boxes and clicking on the *output results* button. The data outputs are stored in the specified *target* folder. The data from individual data points is displayed in a CSV file. Other image data from individual channels that are also displayed include binary masks, NET assessment overlays and graphs indicating increase in area, nuclear deformation as well as DNA:NET ratio.

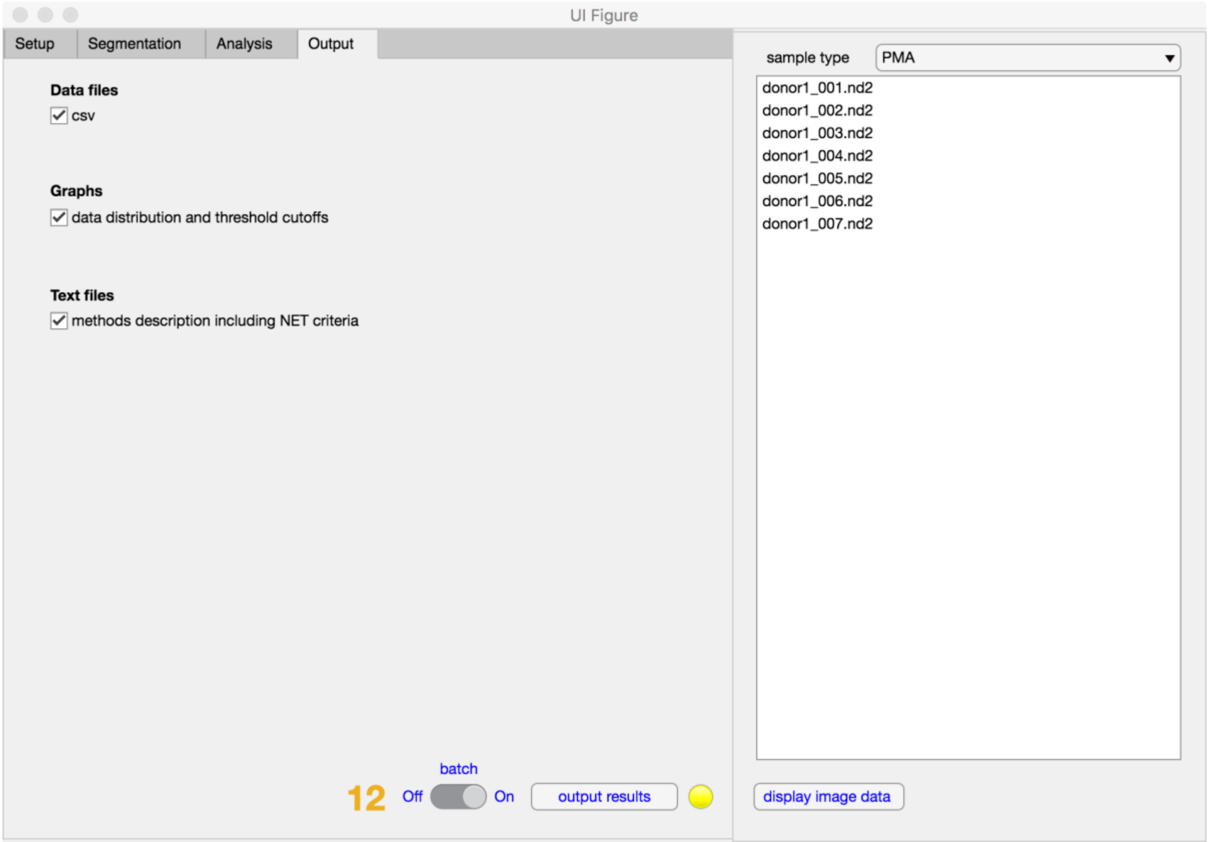

A few examples of the output files are illustrated below. The red vertical line wherever indicated in the graphs depict the threshold.

Figure 1 - Area under NETs in control vs PMA-stimulated neutrophils

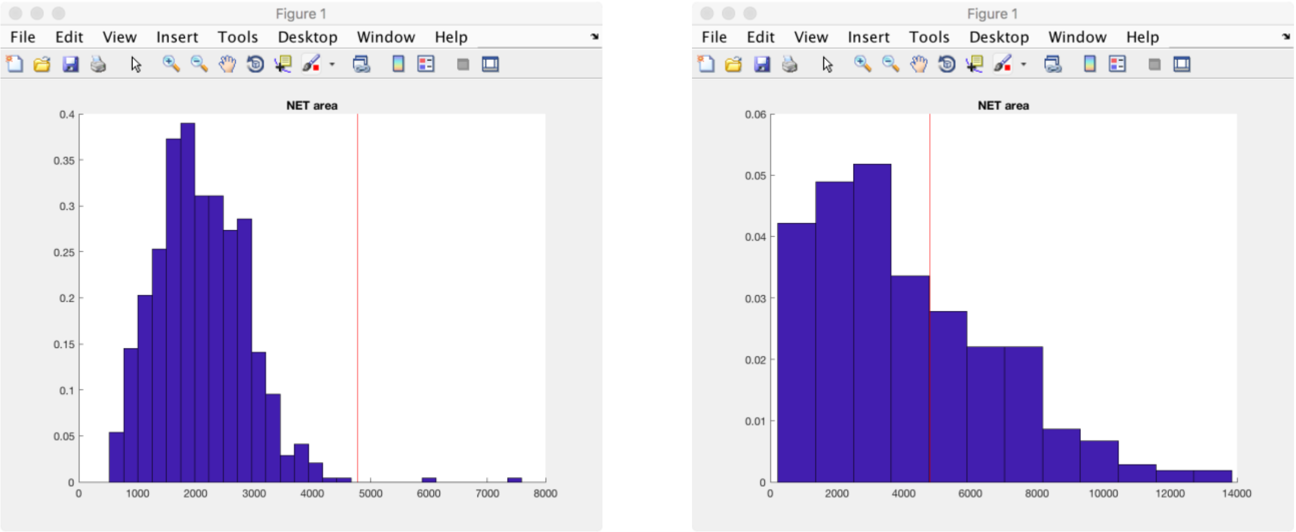

Figure 2 – Nuclear shape in control vs PMA-stimulated neutrophils

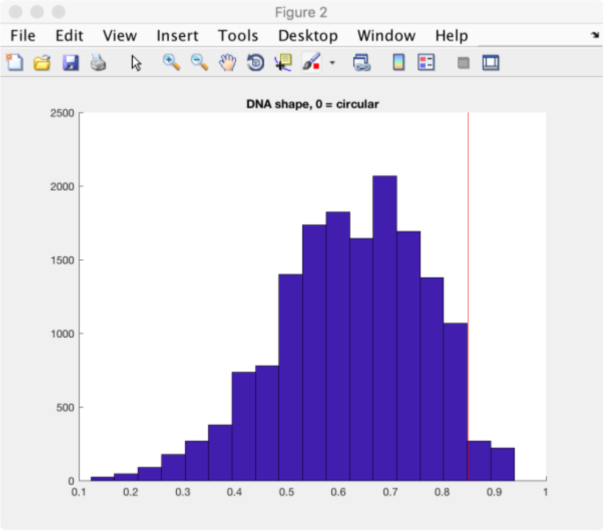

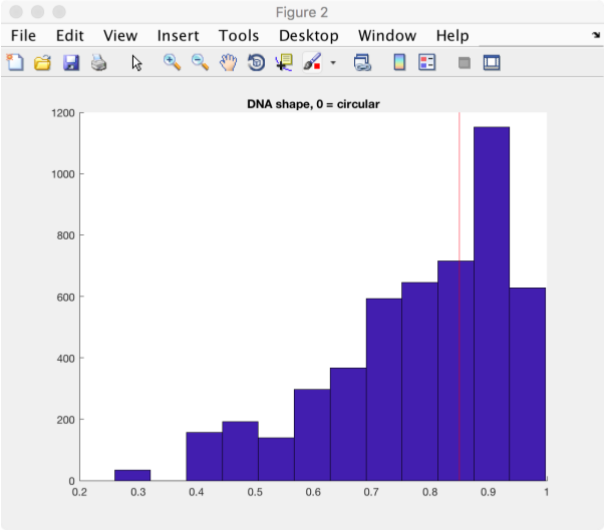

Figure 3 – DNA/NET ratio in control vs PMA-stimulated neutrophils

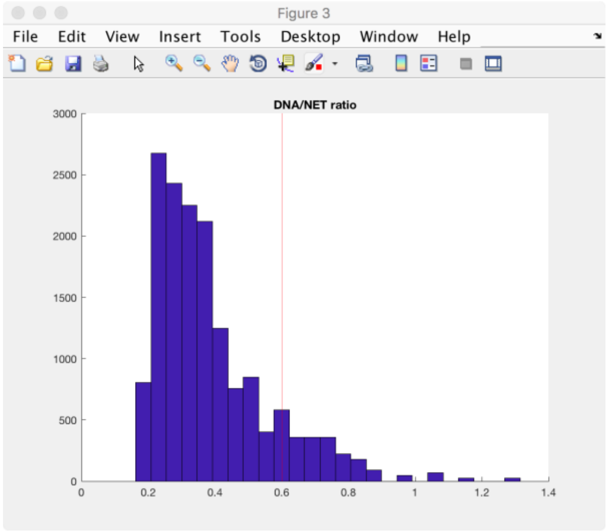

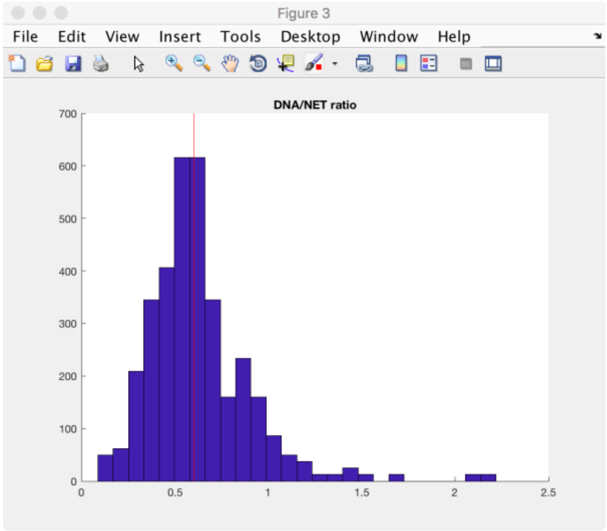

Figure 4 – NET area vs DNA shape in control vs PMA-stimulated neutrophils

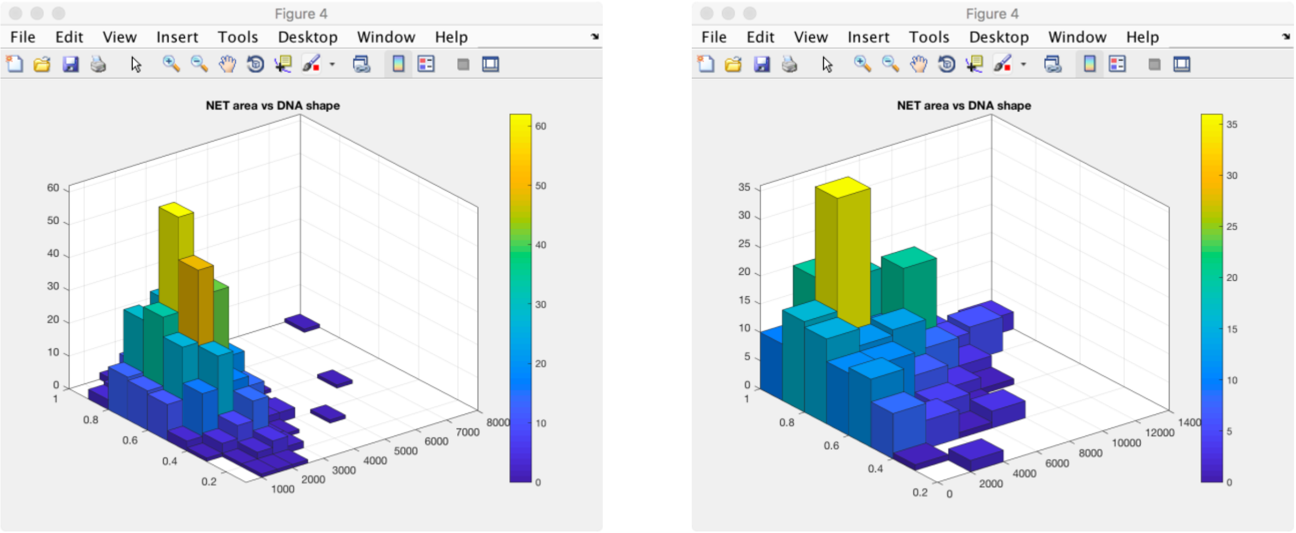

2.13 Loading previous analysis

The data from previously successful runs can be imported into NETQUANT using the *load previous analysis* option.
